# Spatial Distribution and Birth Prevalence of Congenital Heart Disease in Iran: A Systematic Review and Hierarchical Bayesian Meta-analysis

**DOI:** 10.34172/ijhpm.2024.7931

**Published:** 2024-05-07

**Authors:** Roghaye Farhadi Hassankiadeh, Annette Dobson, Somayeh Rahimi, Abdollah Jalilian, Volker J. Schmid, Behzad Mahaki

**Affiliations:** ^1^Department of Biostatistics, School of Health, Kermanshah University of Medical Sciences, Kermanshah, Iran.; ^2^School of Public Health, University of Queensland, Brisbane, QLD, Australia.; ^3^Department of Clinical Biochemistry, Kermanshah University of Medical Sciences, Kermanshah, Iran.; ^4^Department of Statistics, Razi University, Kermanshah, Iran.; ^5^Department of Statistics, LudwigMaximilians-University, Munich, Germany.

**Keywords:** Spatial Distribution, Birth Prevalence, Congenital Heart Disease, Iran, Systematic Review, Hierarchical Bayesian Meta-analysis

## Abstract

**Background:** This study aimed to comprehensively analyze the overall congenital heart disease (CHD) prevalence in live births and children in Iran, along with evaluating the spatial distribution of CHD birth prevalence across various geographical regions within the country.

**Methods:** A Bayesian hierarchical meta-analysis (PROSPERO 2022: CRD42022331281) was performed to determine the pooled prevalence. A systematic search was conducted using Web of Science, ScienceDirect, PubMed, Iranian Research Institute for Information Science and Technology (IranDoc), Scientific Information Database (SID), and Magiran until October 4, 2023. Cross-sectional and cohort studies in both English and Persian languages, focusing on the age range of 0-10 years, were considered for the study population. The study quality was evaluated using the Agency for Healthcare Research and Quality (AHRQ) Risk of Bias tool. Heterogeneity was assessed by I^2^ and τ^2^ statistics, and publication bias by Egger’s and Begg’s tests.

**Results:** The meta-analysis included 62 studies, revealing an overall CHD prevalence of 2.5 per 1000 births. Over time, CHD birth prevalence in Iran has consistently increased. Spatial distribution analysis, including spatial autocorrelation and local spatial autocorrelation, indicated no spatial clustering (*P*=.46) or aggregation (*P*=.65) among Iran’s provinces. Geographic disparities were significant (*P*=.000), with the northern and eastern regions showing the highest and lowest CHD prevalence, respectively.

**Conclusion:** The overall CHD prevalence in Iran is lower than global rates, but it continues to rise. Furthermore, there are variations in birth prevalence among different regions of Iran. Environmental, genetic, socioeconomic, and diagnostic accessibility differences are possibly involved in regional variation. The limitations like heterogeneity among studies, the potential inaccuracy of reports due to limited use of accurate diagnostic methods in some studies, and the absence of population-based models to investigate prevalence, underscore the urgent need for standardized diagnostic approaches, and the utilization of population-wide birth defect registries to accurately assess CHD prevalence in Iran.

## Background

 Congenital anomalies are a series of disorders that occur during embryonic life that could be life-threatening to infants. Congenital heart disease (CHD) accounts for 28% of all birth anomalies and is a significant global health problem.^1^ CHD involves various structural or functional abnormalities affecting the heart or large intrathoracic vessels, which are classified into distinct subtypes based on specific cardiac abnormalities.^2,3^ While CHD is present at birth, it may initially remain clinically silent and only manifest symptoms later in life.^4^ The cardiac lesions are categorized into mild, moderate, and severe.^5^ Most mild lesions do not require intervention or may heal spontaneously. However, severe or critical lesions (25%-35% of CHD) require early intervention or surgery and can be associated with morbidity and mortality.^6^ Early diagnosis through echocardiography and subsequent medical and surgical interventions, have significantly improved the prognosis for severe CHD in developed nations. However, in parts of the developing world, access to treatment for more severe conditions remains unavailable.^7^

 Advancements in medical science have enabled more CHD patients to reach adulthood, presenting a new challenge in the form of grown-up CHD patients and adding to the global health burden.^1^ Early detection of Congenital anomalies reduces the significant economic and social burden on families and society, and elevates the life expectancy of patients. Consequently, identifying cardiac abnormalities during fetal development becomes imperative to mitigate their impact.

 The prevalence of CHD varies among populations, diverse geographic regions, and over time. The most feasible indicator for CHD occurrence is its prevalence per 1000 live births.^8^ In a meta-analysis study, it was estimated that the average global prevalence of CHD in live births, spanning the years 2010 to 2017, was 9.4 per 1000 live births. Notably, Asia exhibited a higher prevalence compared to other global regions during this period.^7^ Furthermore, there is a significant difference in the prevalence of CHD between developed and developing countries.^9^ However, a comprehensive and accurate assessment of CHD prevalence is lacking for numerous countries, including Iran. Iran is a country with ethnic and racial diversity and various geographical regions, there could be inequality in the access and quality of healthcare services. So, it is important to describe the distribution of prevalence of CHD in different geographical areas in this country. Studies conducted in Iran estimate a wide range of prevalence of CHD, with different figures between zero and 26.48 cases per 1000 live births.^10-16^ Gaining insights into the distribution of CHD in each country is imperative for understanding its etiology, identifying high-risk populations, and efficiently implementing targeted services for prevention and management.

 Therefore, we conducted a systematic review and meta-analysis based on available studies up to 2023 to provide a comprehensive nationwide overview of the reported prevalence and spatial distribution of CHD in Iran.

## Methods

 This review protocol was registered on International prospective register of systematic reviews (PROSPERO 2022: CRD42022331281). The Preferred Reporting Items for Systematic Reviews and Meta-Analyses (PRISMA 2020) guideline checklist was followed in this review. We defined PICOST (Population, Intervention [Exposure], Comparison, Outcome, Study design, and Time)^17^ to ensure that the assessment contained only the most relevant studies.

###  Inclusion Criteria

 (1) Participants: This research included studies that investigated the prevalence of CHD among participants from birth to 10 years old. At first, our selection criteria focused on studies investigating CHD prevalence at birth. Upon thorough assessment, the majority of these studies concentrated on the age group of 0-1 years, except for two studies covering broader age ranges of 0-8 years and 0-10 years. These two specific studies were incorporated due to their valuable information into the spatial patterns of CHD prevalence at birth. (2) Study design: Cross-sectional and cohort studies. (3) Language: Studies published in either Persian or English language. (4) Outcomes: The prevalence of CHD was outcome. (5) Publication year: Systematic search was done without any time limit until October 4, 2023.

###  Exclusion Criteria 

 (1) Studies such as reviews, comments, or books. (2) Studies examining the prevalence of CHD in special populations, such as the Down syndrome population, and so on. (3) Studies that reported CHD prevalence exclusively in schoolchildren and adults, without considering the prevalence at birth, were not included.

###  Search Strategies

 We systematically searched the English language databases, including ISI Web of Science, ScienceDirect, PubMed, and Scopus, and Iranian databases, including Iranian Research Institute for Information Science and Technology (IranDoc), Scientific Information Database (SID), Magiran, to identify related and available articles until October 4, 2023. The search strategy involved the utilization of Medical Subject Headings (MeSH) terms, combined with keywords and Boolean operators. The search concepts included terms related to “congenital,” “heart,” “cardiac,” “cardiovascular,” “disease,” “defect,” “anomaly,” “disorders,” “epidemiology,” “prevalence,” “Iran,” and “Iranian.” All articles were searched without any time limit and the search strategy is provided in Supplementary file 1.

###  Data Extraction and Quality Assessment 

 Two authors (RFH and SR) independently conducted the data extraction. The information gathered included details such as the first author, year of publication, study location (province), demographic characteristics (gender), study period, number of patients with CHD used to calculate birth prevalence, total study population, CHD subtype, diagnostic tool, and language of publication (English or Persian). To ensure consistency and reliability, both reviewers worked independently throughout the extraction process, and any discrepancies were resolved through discussion and consensus.

 The quality of the study was assessed using the Agency for Healthcare Research and Quality (AHRQ) Risk of Bias tool.^18^ The use of AHRQ to assess the quality of cross-sectional/prevalence studies has been recommended in several studies.^18-20^ AHRQ tools are also used to assess the quality of cohort studies. All the studies included in this study were cross-sectional, and the cohort study in Iran was not utilized to determine the CHD prevalence. An item was given a score of “0” if the response was “no” or “unclear,” and “1” if the response was “yes.” The maximum scale was 11, and the scores were categorized as low quality (1-3), moderate quality (4-7), and high quality (8-11).^19,21,22^ All of the studies that were included had an AHRQ score, with a range of 7 to 11 (Supplementary file 2).

###  Statistical Methods

 First, a hierarchical Bayesian meta-analysis was conducted to model the log odds (logit) pooled birth prevalence with a 95% confidence interval (CI) (See Supplementary file 3). Then heterogeneity between the studies was examined using the I^2^ index and the τ^2^ test. The I^2^ index is the percentage of effect size variability that is not due to sampling error, and τ^2^ indicates the variation of the true effect sizes. In fixed effect models, τ^2^ = 0 is assumed, which indicates there is no evidence of heterogeneity—threshold values of for heterogeneity of true effect sizes τ^2^: 0.25 (moderate), 0.5 (substantial), 1 (high) and 2 (very high).^23-25^ Also I^2^ > 0.75 was assumed as high-value for heterogeneity.^26,27^

 Subgroup sensitivity analyses were conducted, with a focus on gender, geographic regions, and length of follow-up, to find possible factors of heterogeneity. In addition to Egger’s (test for funnel plot asymmetry) and Begg’s tests, a funnel plot was also used to estimate publication bias. Bayesian hierarchical methods allow for the investigation of between-study heterogeneity and within-study variability. They enable the estimation of the posterior distribution of two parameters (effect and heterogeneity), evaluation of joint and marginal posterior probability distributions, and so on.^28^

 In the time-trend analyses, studies were defined in three-year groups dependent on the year of investigation. The first and second groups included more than 3 years since only three and one study were collected in those periods, respectively. Time trends were plotted using the Moving Average smoothing technique. Factors such as sex, geographic region, and length of follow-up were considered in the subgroup analysis. The overall estimate was then compared to the estimates of the different subgroups using a proportion test. The forest plot showed log(odds) and 95% credible intervals for each study and the pooled results from this meta‐analysis.

###  Spatial Autocorrelation

 Spatial autocorrelation analysis describes how spatial data are interdependent.^29^ The distribution pattern of the birth prevalence of CHD can be expressed as clustered, dispersed, or random countrywide.^30^ Global and local spatial autocorrelations analyses were conducted. To analyze the distribution of CHD prevalence across all provinces, a global spatial autocorrelation method was used. The spatial autocorrelation of province locations and the prevalence of CHD was assessed using the Global Moran’s I method. The range of the Moran index I is -1 to 1. If the data are consistent with the null hypothesis that I = 0, the pattern of distribution can be considered to be random. I > 0 implies that the data is clustered, and I < 0 implies that the data is over-dispersed.^31^ Local spatial autocorrelation was used to investigate the CHD prevalence distribution mode in Iran. Getis-Ord G can be regarded as the index of hot spot analysis and shows that the high or low values are clustered. If G > 0 and Z > 1.96, Iran would be considered a hotspot area, which would indicate that the CHD prevalence distribution inside the Iran was spatially clustered with *P* < .05.^32^

 We fitted the Bayesian hierarchical model and calculated the log odds of CHD birth prevalence. Log odds are symmetric around zero, which makes them easier to work with mathematically.^33^ We then converted these estimates (log odds) back to the prevalence (Supplementary file 3).

###  Software

 Data were analyzed using SPSS 16, MS Excel, ArcGIS 10.2, and R 4.1.3 software. The R packages used included metafor and bayesmeta.^34,35^

## Results

###  Characteristics of the Included Studies

**Figure 1:**
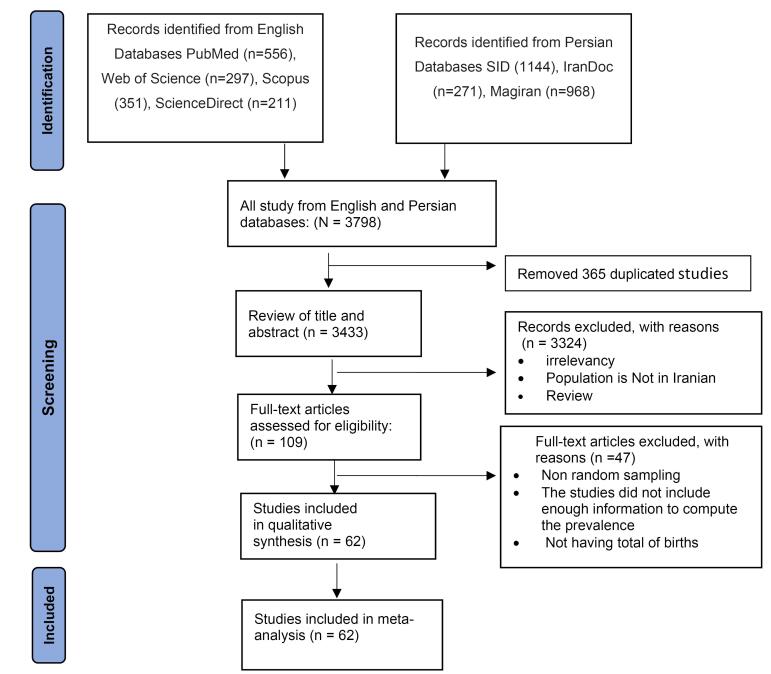
PRISMA Flow Diagram for Selection of Studies. Abbreviations: PRISMA, Preferred Reporting Items for Systematic Reviews and Meta-Analyses; SID, Scientific Information Database; IranDoc, Iranian Research Institute for Information Science and Technology.

 The literature search identified a total of 3798 records for screening. After duplicates were removed, 3433 unique records were screened by titles and abstracts. A total of 109 studies were assessed for full-text eligibility after removing obviously irrelevant articles based on evaluation of their titles and abstracts. Finally, 62 studies remained for further analysis (Supplementary file 4 and Figure 1). These studies included a total of 6 303 428 births and 8989 individuals with CHD.

 Only 20 of the studies explicitly mentioned the use of echocardiography as a diagnostic method, while 4 studies did not specify their diagnostic methods. The remaining studies employed a combination of other diagnostic methods.

**Figure 2:**
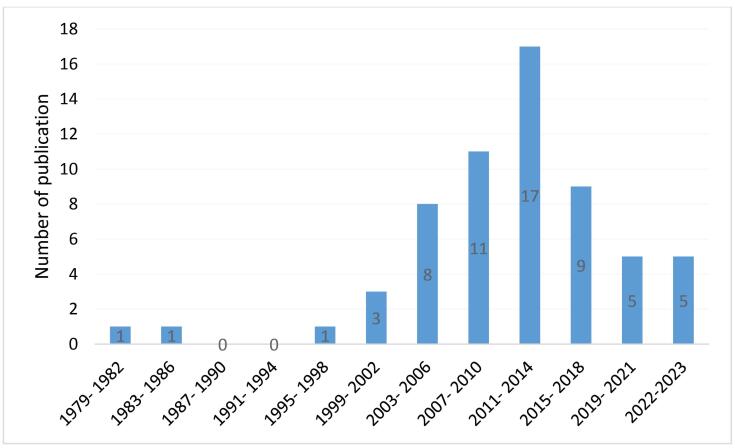
The Number of Studies on the Prevalence of Congenital Heart Disease, Based on the Time of Publication, of 3-Year Groups by 2023.

 As shown in Figure 2, we stratified CHD prevalence studies according to time of publication. From 1979 to 1990, only two studies were available, leading us to categorize these 12 years into one category. For the subsequent years, we categorized studies based on their publication time at 3-year intervals. Most of the studies are related to the years 2011 to 2013, and 38 studies (60%) were reported from 2010.

###  Birth Prevalence of Congenital Heart Disease Over Time

**Figure 3:**
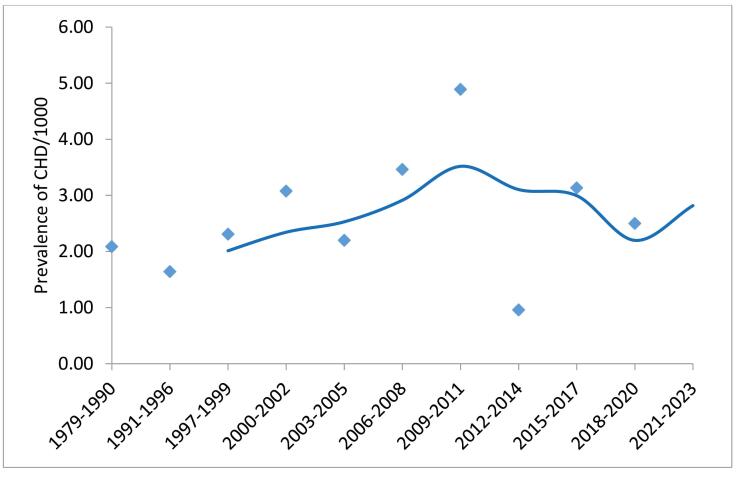
Iranian Overall Birth Prevalence of Congenital Heart Disease Over Time. Timeline of the reported birth prevalence of all CHD from 1979 to 2023. The squares show each period’s calculated birth prevalence values, while the blue line depicts the time trend. Abbreviation: CHD, congenital heart disease.

 Based on the results of the Bayesian hierarchical meta-analysis, the birth prevalence of CHD in Iran from (years of publication) 1979-2023 was 2.50 (95% CI: 1.83, 3.37) per 1000. Figure 3 displays the time-trend analyses of CHD prevalence using a simple moving average with a 3-interval. This figure represents a general pattern of increase over the last decades. The highest prevalence was in 2012-2014 (4.89; 95% CI = 2.81 to 8.27 per 1000). The heterogeneity of log (odds) between studies was observed (I^2^= 99.4%; τ^2^ = 1.26, 95% CI = 1.1 to 1.5).

###  Birth Prevalence of CHD in Different Geographical Regions

**Figure 4:**
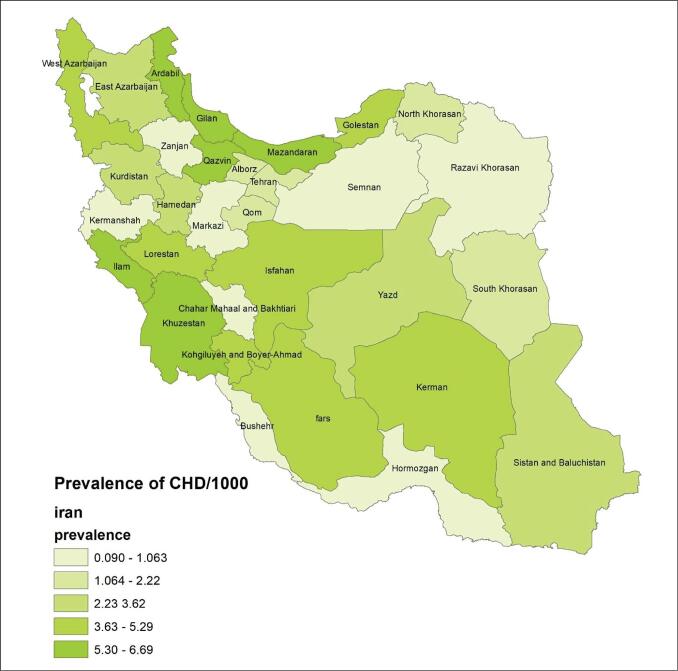
Geographical Distribution of Prevalence of Congenital Heart Disease at the Province Level in Iran During 1979-2023. Abbreviation: CHD, congenital heart disease.

 Remarkable geographical variations were observed in the total prevalence of CHD (χ^2^ = 34516, *df* = 4, *P* =.000). Figure 4 shows the pooled prevalence of CHD in various provinces of Iran using the classification based on geometrical intervals. The highest birth prevalence was found in Ilam, Mazandaran and Ardabil (6.69, 6.63, and 6.43, per 1000, respectively) and were shown on the map in dark green. The lowest were found in Hormozgan (0.09 per 1000) and was shown on the map in light green (Figure 4 and Table 1). It is worth noting that no studies were found in the three provinces. To estimate these provinces, we categorized Iran’s provinces into five regions: North, East, Central, West, and South. We then conducted a meta-analysis by grouping these regions to determine the prevalence of CHD in each region. Finally, we used this information to estimate the spatial distribution of CHD prevalence by substituting the corresponding prevalence for each of the three provinces. The overall estimated CHD prevalence in North, East, West, South, and Central regions was 5.17, 1.33, 2.35, 4.73, and 2.11 per 1000, respectively (See Supplementary file 5, Table S4).

**Table 1:** Total Congenital Heart Disease Birth Prevalence of Various Provinces of Iran

**Geographical Regions**	**No. of Studies**	**Pooled Prevalence**	**Lower bound of Pooled Prevalence (95% CI)**	**Upper Bound of Pooled Prevalence (95% CI)**	**Ref**.
Alborz	3	2.19	0.80	4.82	^36-38^
Ardebil	2	6.43	0.79	26.08	^39,40^
Bushehr	1	0.82	0.00	14.77	^41^
Chahar Mahaal and Bakhtiari	1	0.2	0.01	3.68	^42^
East Azarbayejan	8	2.46	1.71	3.53	^43-50^
Fars	1	4.63	1.09	19.46	^51^
Gilan	1	5.54	1.68	18.71	^52^
Golestan	6	4.73	1.89	9.29	^53-57^
Hamadan	0 (Estimated)	2.35	1.63	3.95	-
Hormozgan	1	0.09	0.01	1.66	^58^
Ilam	1	6.69	1.49	29.89	^59^
Isfahan	5	4.07	1.49	11.21	^60-64^
Kerman	1	4.45	1.47	13.93	^65^
Kermanshah	1	3.37	1.13	10.25	^66^
Khorasan-e-Razavi	2	0.66	0.16	1.93	^67,68^
Khuzestan	3	5.62	3.91	7.77	^69-71^
Kohgiluyeh and Boyer-Ahmad	0 (Estimated)	4.73	3.05	7.03	-
Kurdistan	0 (Estimated)	2.35	1.63	3.95	-
Lorestan	1	4.12	1.41	12.44	^72^
Markazi	1	0.25	0.01	3.33	^10^
Mazandaran	1	6.63	2.03	21.88	^73^
North Khorasan	1	1.07	0.33	3.61	^68^
Qazvin	1	6.08	2.09	18.34	^74^
Qom	1	1.95	0.64	6.12	^75^
Semnan	1	0.15	0.03	0.69	^76^
Sistan and Baluchistan	1	0.34	0.02	6.06	^77,78^
South Khorasan	2	1.59	0.86	3.02	^79,80^
Tehran	10	2.09	0.86	4.97	^16,36-38,75,81-85^
West Azarbayejan	1	4.51	0.50	24.36	^86^
Yazd	5	2.96	1.73	4.87	^87-90^
Zanjan	1	0.39	0.13	1.25	^91^

Abbreviation: CI, confidence interval. *Note:* The study of two provinces of Alborz and North Khorasan overlaps with Tehran and Khorasan-e-Razavi provinces, and three provinces were estimated to be neighboring provinces (refer to the limitations section).

###  Spatial Analysis

 To obtain an estimate of the spatial distribution of the prevalence of CHD, global spatial autocorrelation was estimated using Moran’s I. The Moran’s I test result showed no spatial clustering in the distribution of CHD prevalence in various provinces (I = -0.11, *P* = .46). Similarly, for local spatial autocorrelation, the Getis G statistic was not statistically significant different from zero (*P* = .65), indicating no evidence of spatial aggregation (Supplementary file 5, Figure S1).

###  Birth Prevalence of Congenital Heart Disease by Gender

 Although gender is not reported in all studies, a total of 12 studies (242 970 births) identified the birth prevalence of total CHD in different gender, including 720 male and 585 female patients.^16,54,56,60,62,72,74,75,79,84,89,92^ In males, the overall CHD prevalence was 2.9 (95% CI = 1.5 to 5.5), while in females the birth prevalence was 2.4 (95% CI = 1.3 to 4.73) (χ^2^ = 45.472, *df* = 1, *P* = .000).

###  Birth Prevalence of Congenital Heart Disease by Length of Follow-up

 The length of follow-up of included studies was divided into less than 12 months (9 studies), between 12 and 24 months (26 studies), and more than 24 months follow-up (27 studies). The duration or length of the follow-up study in this investigation refers to the period during which all infants born were examined to identify those with congenital heart malformations. Length of follow up analyses indicated a rise from 0.71 per 1000 in studies with less than 12 months, to 3.05 per 1000 in studies with more than 24 months follow-up (*P* < .001) (Table 2).

**Table 2:** Iranian Overall Birth Prevalence of CHD/1000 Over the Course Length of Follow up

**Length of Follow-up**	**Pooled Birth Prevalence Per 1000 (95% CI)**
Less than 12 months	0.71 (0.34, 1.47)
12 to 24 months	1.9 (1.1, 3.2)
More than 24 months	3.05 (1.78, 5.02)

Abbreviations: CI, confidence interval; CHD, congenital heart disease.

###  Birth prevalence of major subtypes of CHD

 The birth prevalence of subtypes reported in the studies is presented in Table 1. Only 17 studies (involving 4569 individuals with CHD and 823 333 births) focused on identifying the CHD prevalence and its most common subtypes.^16,39,44,54-57,60-62,70,72,78,84,89,92,93^ The rest of studies had a more general focus on the prevalence of congenital anomalies. The total pooled birth prevalence of CHD for studies that reported CHD subtype was 5.71 per 1000 births (95% CI= 3.51 to 9.29) (Supplementary file 5, Figure S2 and Table S4).

 The subtype’s prevalence with 95% confidence intervals was calculated for these 17 studies (Table 3). A Bayesian meta-analysis was used to calculate the pooled birth prevalence, considering the events of each subgroup and the total number of births in the meta-analysis formula. However, when calculating the percentages, the total number of CHDs was used instead of the total number of births. The most frequent types of CHD were found to be ventricular septal defect (VSD) (19.78% of total prevalence), atrial septal defect (ASD) (16.11% of total birth prevalence), and patent ductus arteriosus (PDA) (12.56% of total birth prevalence), respectively. In total, the first six subtypes included 59.30% of the total burden of CHD.

**Table 3:** The Birth Prevalence and Percentage of Specific Congenital Heart Disease Subtypes

**Major Subtypes of CHD**	**Abbreviations**	**No. of Studies**	**Pooled Birth Prevalence Per 1000 (95% CI)**	**Percentage of Birth Prevalence Subtypes, % (95% CI)**
Ventricular septal defect	VSD	14	1.17 [0.6, 2.44]	19.78 [12.78, 29.11]
Atrial septal defect	ASD	13	0.91 [0.51, 1.56]	16.11 [11.05, 21.59]
Patent ductus arteriosus	PDA	12	0.72 [0.41, 1.2]	12.56 [10.43, 14.93]
Tetralogy of Fallot	TOF	14	0.35 [0.17, 0.69]	6.00 [3.42, 9.98]
Transposition of the great arteries	TGA	7	0.24 [0.1, 0.55]	3.9 [1.95, 6.85]
Pulmonary stenosis	PS	9	0.22 [0.12, 0.38]	3.14 [1.93, 5.17]
Coarctation of aorta	CoA	4	0.19 [0.08, 0.4]	2.96 [1.48, 5.37]
Pulmonary atresia	PA	4	0.13 [0.06, 0.26]	2.19 [1.25, 3.39]
Tricuspid regurgitation	TR	4	0.12 [0.046, 0.3]	1.85 [0.78, 4.07]
Aortic stenosis	AS	5	0.10 [0.05, 0.18]	1.34 [0.84, 2.77]
Hypoplastic left heart syndrome	HLHS	2	0.08 [0.03, 0.2]	1.25 [0.55, 2.5]
Mitral regurgitation	MR	2	0.07 [0.03, 0.13]	0.92 [0.46, 1.83]
Atrioventricular septal defect	AVSD	4	0.06 [0.04, 0.1]	0.89 [0.53, 1.52]
Mitral valve prolapse	MVP	3	0.05 [0.03, 0.11]	0.78 [0.37, 1.6]
Patent foramen ovale	PFO	3	0.05 [0.03, 0.11]	0.78 [0.37, 1.6]
Pulmonary hypertension	PH	1	0.05 [0.02, 0.11]	0.78 [0.34, 1.75]
Pulmonary regurgitation	PR	1	0.05 [0.02, 0.11]	0.78 [0.34, 1.75]

Abbreviations: CI, confidence interval; CHD, congenital heart disease.

###  Heterogeneity, Subgroup Analyses, Publication Bias, and Sensitivity Analysis 

 In our study, within the pooled prevalence for subgroups defined by gender, geographic region, length of follow-up, and major subtypes of CHD, the heterogeneity indices for the birth prevalence of CHD were high (I^2^ > 75%, τ^2^ > 1) (See heterogeneity plot in Figure S3 and Table S4). Publication bias was not statistically significant in the meta-analysis (*P *value of Begg’s test = .97, *P* value of Egger’s test < .0001) (See funnel plot and in Supplementary file 5, Figure S4). Moreover, the robustness of the Bayesian analysis was verified with various sensitivity analyses. Sensitivity analyses using several weakly informative prior distributions demonstrated the robustness of these selections.

## Discussion

 In this study, our aim was to calculate the birth prevalence of CHD in Iran and investigate its geographical distribution. Our meta-analysis included 62 studies, revealing an overall CHD prevalence of 2.5 per 1000 births. This estimate of CHD prevalence was lower than the global prevalence (8.2 per 1000) and the Asian prevalence (9.3 per 1000).^1,7^ This difference can likely be attributed to a combination of the design of the main studies, the selection of the studied population, various diagnostic methods, environmental and genetic influences, socio-economic factors, and differences in monitoring models (hospital-based/ population-based).^32^ All studies included in our meta-analysis were hospital-based monitoring models. Zhao et al, in their meta-analysis, revealed that population-based monitoring models showed a significantly higher prevalence of CHD compared to hospital-based models. In their predominantly hospital-based study, the overall prevalence of CHD in China was estimated to be 2.5 per 1000, consistent with the findings of our study.^32^

 In studies specifically focusing on CHD and reporting its subtypes (17 studies), the estimated total birth prevalence of CHD was 5.94 (95% CI = 3.47 to 10.83) per 1000 births. Notably, 71% of these studies employed echocardiography, which enhances the capability to detect minor defects compared to physical examination. It is worth mentioning that, in the majority of the 62 studies where we calculated the overall prevalence, the diagnosis was based on physical examination.^1,94^ Consequently, the calculated overall prevalence of CHD in Iran might be underestimated. The diagnostic method employed plays a pivotal role in estimating prevalence rates, emphasizing the urgent need to enhance skills and awareness within the healthcare system for accurate CHD diagnosis. Achieving this goal involves increasing the presence of medical specialists and improving access to advanced diagnostic tools, particularly echocardiography. In addition, the use of more accessible and cost-effective methods such as pulse oximetry can also help in the early detection of CHD cases.^93,95^

 Furthermore, 40% of the studies were conducted before 2010. This suggests that we need new studies with more accurate study designs.^93,95^ Moreover, the conducted studies did not include data on the number of cases that did not survive due to severe heart disease during the fetal and neonatal periods. Additionally, pregnancy screening and evaluations of aborted fetuses may increase the frequency. So, by addressing these factors, we can improve the accuracy of CHD prevalence estimates.

 In our study, spatial autocorrelation (global and local autocorrelation) showed that there was no spatial clustering across provinces and no aggregation characteristics within provinces in the overall birth prevalence of CHD. On the other hand, significant geographic differences were identified. Compared with other regions, the northern and eastern regions had the highest and lowest CHD prevalence, respectively. While there is no clear explanation, the difference could be influenced by various environmental, genetic factors, socioeconomic factors, years of study, and variation in the methodologies employed to assess CHD prevalence across different provinces of Iran.^7,54^ Also, due to an inadequate healthcare system and follow-up, lack of accessible diagnostic tools, CHD prevalence may be underestimated in some studies and provinces.^96^ It should be noted that only 6 eligible studies were related to eastern Iran, which all studies were published before 2014. Also, in these studies, the diagnostic tool was not reported exactly and echocardiography was not used for diagnosis.

 We also found that the CHD birth prevalence was higher in males versus females, which is consistent with some previous studies.^19,97^ The precise processes underlying males’ higher CHD birth prevalence remain unknown and deserve additional investigation.^98^

 Subgroup analysis on the length of follow-up indicated that studies with a length of follow-up greater than 24 months had a significantly greater birth prevalence of CHD. One of the reasons for the heterogeneity was the length of follow-up, which is also mentioned in the study by Zhao et al.^32^

 The overall reported birth prevalence of CHD has increased over time, rising from 1.58 per 1000 births in 1979-1990 to 2.5 per 1000 births in 2021-2023, respectively. It seems that industrialization and urbanization have an effect on the occurrence of CHD, indeed, the increase of environmental risk factors (air quality, organic solvents, groundwater pollution, etc) over time may increase the risk of congenital heart defects.^32,99^ On the other hand, in recent years, due to the improvement of diagnostic and screening methods, the diagnosis of CHD has increased.^10^ This may lead to an increase in the birth prevalence of CHD and specific subtypes such as VSD, ASD, PDA, tetralogy of Fallot, pulmonary stenosis, and transposition of the great arteries over time.^100^ The results of our study also showed that these six common subtypes constitute 59.30% of the total burden of CHD.

###  Policy Implications

 There are two main implications of the current findings for health policy and data collection. First, while following regional estimates, country-specific criteria can be utilized to plan and implement health intervention programs to address and reduce the burden of congenital abnormalities. These programs must take into account the rise in healthcare costs brought on by congenital defects, particularly in terms of long-term care. Second, analysis of data coverage revealed that there are fewer health records available for newborns and certain gestational periods. So, these results will help create a database for subsequent research on the etiology and racial, ethnical and environmental components differences in CHD in the region. The results can inform important modifications in health policies for the enhancement of therapeutic and diagnostic capabilities.^16,54^

###  Strengths and Limitations of This Study

 This study has several strengths. First, this meta-analysis is the first to systematically collect published studies on the spatial and total birth prevalence of CHD in Iran. We used all the studies in which the birth prevalence of CHD was reported. Therefore, it has added knowledge about the prevalence of the disease and spatial distribution of the Iranian population by examining data from 31 provinces and 62 studies and has identified the major subtype’s birth prevalence. Second, we conducted a Bayesian hierarchical meta‐analysis to calculate a pooled CHD prevalence. The Bayesian hierarchical random effect model is an alternative to the classical meta-analysis for estimating the precise pooled effect size, particularly when there are few studies. In this study, subgroups such as CHD subtypes were identified in only a few studies. Forth, in the Bayesian hierarchical meta-analysis, the credible interval is slightly wider than in the classical meta-analysis, and the results will be more reliable. As a result, compared to classical meta-analysis, the Bayesian hierarchical meta-analysis is more conservative and more consistent.^101^

 This review has substantial limitations. First, there was significant heterogeneity in our review. Although subgroup analyses were performed by geographic regions, periods, and length of follow-up, there is still significant heterogeneity between subgroups. On the other hand, heterogeneity was unavoidable because of the difference in the design of original studies, selection of study population, different diagnosis methodologies, physician abilities among participating hospitals, maternal age, and gestational age.^32,102^ Second, some studies did not have annual prevalence data. For instance, they reported the prevalence for the entire period from 2004 to 2012 and separately for the years 2004 to 2006, 2007 to 2009, and 2010 to 2012. So, we adopted the strategy that this study could have been counted three times when performing the time trend analysis. This may have had a minimal effect on the results of the time trend analysis.^32^ Third, because there is not sufficient data in some province and some years, the result should be interpreted with caution. For instance, in the administrative divisions of Iran, the number of provinces has increased from 28 to 31 in the last two decades, so we equated the prevalence of new provinces for which we did not have with the prevalence of the provinces that previously belonged to the same area (2 provinces). On the other hand, no study was found for three provinces. Therefore, based on geographical regions, we divided Iran’s provinces into 5 regions: North, East, Central, West, and South. We performed a meta-analysis by sub-grouping these 5 regions and calculated the prevalence of each region, then substituted the corresponding prevalence of these provinces to obtain the spatial distribution of CHD prevalence.

## Conclusion

 The overall prevalence of CHD in Iran is lower than the global rate, but it has generally increased from 1975 to 2023. Furthermore, there were significant geographical differences among the different regions of Iran. This observation can likely be attributed to factors such as various environmental and genetic, socio-economic, and availability of healthcare and diagnostic facilities that should be measured or controlled in future studies. Moreover, while the total number of studies was adequate, the prevalence of CHD was found to be scattered and sparse in some provinces and some years. Therefore, it is imperative to develop comprehensive population-based prospective birth defect registries that encompass the entire population of Iran to accurately ascertain the prevalence of CHD.

## Ethical issues

 Not applicable.

## Competing interests

 Authors declare that they have no competing interests.

## Disclaimers

 The views presented in this article are those of the authors.

## Supplementary files


Supplementary file 1. Search Strategy in the Mentioned Databases.

Supplementary file 2. Quality Assessment (ARHQ Methodology) of Checklist for Cross-sectional/Prevalence Study.

Supplementary file 3. Bayesian Hierarchical Pooling of CHD Birth Prevalence.

Supplementary file 4 contains Tables S1-S3.

Supplementary file 5 contains Figures S1-S4 and Table S4.

## References

[1] van der Linde D, Konings EE, Slager MA (2011). Birth prevalence of congenital heart disease worldwide: a systematic review and meta-analysis. J Am Coll Cardiol.

[2] Mitchell SC, Korones SB, Berendes HW (1971). Congenital heart disease in 56,109 births Incidence and natural history. Circulation.

[3] Fahed AC, Gelb BD, Seidman JG, Seidman CE (2013). Genetics of congenital heart disease: the glass half empty. Circ Res.

[4] Brickner ME, Hillis LD, Lange RA (2000). Congenital heart disease in adults Second of two parts. N Engl J Med.

[5] Hoffman JI, Kaplan S (2002). The incidence of congenital heart disease. J Am Coll Cardiol.

[6] Miranović V (2016). The incidence of congenital heart defects in the world regarding the severity of the defect. Vojnosanit Pregl.

[7] Liu Y, Chen S, Zühlke L (2019). Global birth prevalence of congenital heart defects 1970-2017: updated systematic review and meta-analysis of 260 studies. Int J Epidemiol.

[8] Mason CA, Kirby RS, Sever LE, Langlois PH (2005). Prevalence is the preferred measure of frequency of birth defects. Birth Defects Res A Clin Mol Teratol.

[9] Wu W, He J, Shao X (2020). Incidence and mortality trend of congenital heart disease at the global, regional, and national level, 1990-2017. Medicine (Baltimore).

[10] Shahmohammadi F, Ahmadi MA. Statistical investigation of the gross congenital anomalies in alive newborns in Taleghani hospital–Arak. J Arak Uni Med Sci 1997;1(4):23-29. [Persian].

[11] Daliri S, Sayehmiri K, Asadollahi K, Rezaei N, Saroukhani D, Karimi A (2018). Prevalence of congenital anomalies in Iran: a systematic review and meta-analysis. Iran J Neonatol.

[12] Irani M, Khadivzadeh T, Asghari Nekah SM, Ebrahimipour H, Tara F (2018). The prevalence of congenital anomalies in Iran: a systematic review and meta-analysis. Iran J ObstetGynecolInfertil.

[13] Vatankhah S, Jalilvand M, Sarkhosh S, Azarmi M, Mohseni M (2017). Prevalence of congenital anomalies in Iran: a review article. Iran J Public Health.

[14] Zahed Pasha Y, Vahedi A, Zamani M, Alizadeh-Navaei R, Zahed Pasha E (2017). Prevalence of birth defects in Iran: a systematic review and meta-analysis. Arch Iran Med.

[15] Siabani H, Siabani S, Gholizadeh L (2019). Epidemiology of congenital heart defects in Iran: a systematic review. Online J Cardiol Res Rep.

[16] Bagheri MM, Torabi-Nezhad MH, Jamali Z, Baneshi MR. Prevalence and etiology of heart murmurs in 2-24-months-old infants Kerman, Iran. J Kerman Univ Med Sci 2014;21(4):114-122. [Persian].

[17] Sarri G, Patorno E, Yuan H (2022). Framework for the synthesis of non-randomised studies and randomised controlled trials: a guidance on conducting a systematic review and meta-analysis for healthcare decision making. BMJ Evid Based Med.

[18] Xu J, Ji Z, Zhang P, Chen T, Xie Y, Li J (2023). Disease burden of COPD in the Chinese population: a systematic review. Ther Adv Respir Dis.

[19] Zeng X, Zhang Y, Kwong JS (2015). The methodological quality assessment tools for preclinical and clinical studies, systematic review and meta-analysis, and clinical practice guideline: a systematic review. J Evid Based Med.

[20] Rostom A, Dubé, Cranney A, et al. 104 Celiac disease: summary. In: AHRQ Evidence Report Summaries. Rockville, MD: Agency for Healthcare Research and Quality (US); 2004.

[21] Mamikutty R, Aly AS, Marhazlinda J (2021). Selecting risk of bias tools for observational studies for a systematic review of anthropometric measurements and dental caries among children. Int J Environ Res Public Health.

[22] Ma LL, Wang YY, Yang ZH, Huang D, Weng H, Zeng XT (2020). Methodological quality (risk of bias) assessment tools for primary and secondary medical studies: what are they and which is better?. Mil Med Res.

[23] Martel M, Negrín MA, Vázquez-Polo FJ (2023). Bayesian heterogeneity in a meta-analysis with two studies and binary data. J Appl Stat.

[24] Spiegelhalter DJ, Abrams KR, Myles JP. Bayesian Approaches to Clinical Trials and Health-Care Evaluation. Vol 13. John Wiley & Sons; 2004.

[25] Harrer M, Cuijpers P, Furukawa TA, Ebert DD. Doing Meta-Analysis with R: A Hands-On Guide. 1st ed. Boca Raton, FL: Chapman & Hall, CRC Press; 2021. doi:10.1201/9781003107347.

[26] Huedo-Medina TB, Sánchez-Meca J, Marín-Martínez F, Botella J (2006). Assessing heterogeneity in meta-analysis: Q statistic or I2 index?. Psychol Methods.

[27] Mostafaei S, Keshavarz M, Sadri Nahand J (2020). Viral infections and risk of thyroid cancer: a systematic review and empirical Bayesian meta-analysis. Pathol Res Pract.

[28] Gelman A, Carlin JB, Stern HS, Rubin DB. Bayesian Data Analysis. Chapman & Hall, CRC Press; 1995.

[29] Ejigu BA, Wencheko E, Berhane K (2018). Spatial pattern and determinants of anaemia in Ethiopia. PLoS One.

[30] Ma J, Gao X, Liu B, Chen H, Xiao J, Wang H (2019). Epidemiology and spatial distribution of bluetongue virus in Xinjiang, China. PeerJ.

[31] Salima BA, Bellefon MD. Spatial autocorrelation indices. In: Handbook of Spatial Analysis: Theory Aplication with R. European Statistical System (ESS). 2018:51-68.

[32] Zhao L, Chen L, Yang T (2020). Birth prevalence of congenital heart disease in China, 1980-2019: a systematic review and meta-analysis of 617 studies. Eur J Epidemiol.

[33] Albert J, Hu J. Probability and Bayesian Modeling. CRC Press; 2019.

[34] Röver C. Bayesian random-effects meta-analysis using the bayesmeta R package. ArXiv [Preprint]. November 23, 2017. Available from: https://arxiv.org/abs/1711.08683.

[35] Viechtbauer W (2010). Conducting meta-analyses in R with the metafor package. J Stat Softw.

[36] Zamani A, Amini E, Kaveh M, Aminzadeh V. Prevalence of congenital malformations in neonate born in Imam Khomeini and Shariati hospitals. Sci J Forensic Med 2000;6(20):19-25. [Persian].

[37] Akbari M, Sobhani A, Ragerdi Kashani I, Amini E, Rezai Z, Shajari H. Incidence of observable congenital abnormalities among births: Mirza Kochak-Khan, Imam Khomeini and Shariati hospitals (November 2000 to September 2001). Tehran Univ Med J 2003;61(3):212-216. [Persian].

[38] Shajari H, Mohammadi N, Karbalai Aghai M. Prevalence of congenital malformations observed in neonates in Shariati hospital (1381-1383). Iran J Pediatr 2006;16(3):308-312. [Persian].

[39] Mirzarahimi M, Saadati H, Doustkami H, Alipoor R, Isazadehfar K, Enteshari A (2011). Heart murmur in neonates: how often is it caused by congenital heart disease?. Iran J Pediatr.

[40] Alijahan R, Mirzarahimi M, Ahmadi Hadi P, Hazrati S (2013). Prevalence of congenital abnormalities and its related risk factors in Ardabil, Iran, 2011. Iran J ObstetGynecolInfertil.

[41] Pouladfar G, Mallahzadeh A. The prevalence of minor congenital anomalies and normal variations in neonates in Bushehr port. Iran South Med J 2005;8(1):43-52. [Persian].

[42] Sereshti M, Kazemian A, Banaeian S. Prevalence of apparent major congenital malformations and some associated factors, in terminated pregnancies in Hajar hospital of Shahrekord, 2005-2006, Iran. J Shahrekord Univ Med Sci 2008;10(1):36-43. [Persian].

[43] Dastgiri S, Imani S, Kalankesh L, Barzegar M, Heidarzadeh M (2007). Congenital anomalies in Iran: a cross-sectional study on 1574 cases in the north-west of country. Child Care Health Dev.

[44] Dastgiri S, Taghizadeh M, Heidarzadeh M (2011). Early diagnosis and screening of congenital cardiac anomalies. Cardiol Young.

[45] Samadirad B, Khamnian Z, Hosseini MB, Dastgiri S (2012). Congenital anomalies and termination of pregnancy in Iran. J Pregnancy.

[46] Mashhadi Abdolahi H, Kargar Maher MH, Afsharnia F, Dastgiri S (2014). Prevalence of congenital anomalies: a community-based study in the northwest of Iran. ISRN Pediatr.

[47] Rostamizadeh L, Bahavarnia SR, Gholami R (2017). Alteration in incidence and pattern of congenital anomalies among newborns during one decade in Azarshahr, northwest of Iran. Int J Epidemiol Res.

[48] Stone DH, Dastgiri S, Heidarzadeh M, Mashhadi Abdolahi H, Imani S, Kargar Maher MH (2017). Uses, limitations, and validity of a registry of congenital anomalies in Iran: a critical review. J Environ Public Health.

[49] Molapour H, Dastgiri S, Davtalab Esmaeili E. Prevalence and time trend of congenital heart defects: a registry-based study in Iran. medRxiv [Preprint]. December 17, 2021. Available from: https://www.medrxiv.org/content/10.1101/2021.12.16.21267826v1.

[50] Tarighat F, Golshan E, Dastgiri S. Prevalence of congenital anomalies in the northwest of Iran. Depiction of Health 2021;12(4):417-425. [Persian].

[51] Vafaei H, Rafeei K, Dalili M, Asadi N, Seirfar N, Akbarzadeh-Jahromi M (2021). Prevalence of single umbilical artery, clinical outcomes and its risk factors: a cross-sectional study. Int J Reprod Biomed.

[52] Jalali SZ, Fakhraie SH, Afjaei SA, Kazemian M (2015). The incidence of obvious congenital abnormalities among the neonates born in Rasht hospitals in 2011. J Kermanshah Univ Med Sci.

[53] Golalipour MJ, Ahmadpour-Kacho M, Vakili MA (2005). Congenital malformations at a referral hospital in Gorgan, Islamic Republic of Iran. East Mediterr Health J.

[54] Nikyar B, Sedehi M, Mirfazeli A, Qorbani M, Golalipour MJ (2011). Prevalence and pattern of congenital heart disease among neonates in Gorgan, northern Iran (2007-2008). Iran J Pediatr.

[55] Golalipour MJ, Mirfazeli A, Mobasheri E (2013). Incidence and pattern of congenital malformations in Gorgan-north of Iran. J Med Sci.

[56] Nikyar B, Sedehi M, Qorbani M, Nikyar A, Golalipour MJ (2014). Ethnical variations in the incidence of congenital heart defects in Gorgan, northern Iran: a single-center study. J Tehran Heart Cent.

[57] Mirfazeli A, Kaviany N, Hosseinpoor K, Aryaie M, Golalipour MJ (2018). Birth defects in northern Iran (2008-2013). Iran J Public Health.

[58] Nazemi Gheshmi A, Nikuei P, Khezri M, et al. The frequency of congenital anomalies in newborns in two maternity hospitals in Bandar Abbas: 2007-2008. Genetics in the Third Millennium 2012;9(4):2554-9. [Persian].

[59] Sayehmiri K, Kaffashian MR, Ranaei E (2016). Investigating the prevalence of congenital anomalies and its associated factors in Ilam city. J Basic Res Med Sci.

[60] Movahedian AH, Noorbakhsh SE, Mosaiebi Z, Mazoochi T, Moosavi SG. Prevalence of congenital heart disorders in neonates hospitalized in Shahid Beheshti hospital during the years 1996-2000. Feyz 2001;5(2):76-80. [Persian].

[61] Mosayebi Z, Movahedian AH (2007). Pattern of congenital malformations in consanguineous versus nonconsanguineous marriages in Kashan, Islamic Republic of Iran. East Mediterr Health J.

[62] Movahedian AH, Heidarzadeh M, Mosayebi Z, Soleimani Z, Sayyah M, Kadkhodaii J (2017). Congenital heart disease: frequency and the need for intervention on the first year of birth. J Res Med Dent Sci.

[63] Davari HA, Javanmardi Z, Hooshangi Z, Moazem E (2019). Major congenital anomalies and associated risk factors in Isfahan province, Iran, 2014-2015. J Isfahan Med Sch.

[64] Saberi M, Hosseinpour M, Khaleghnejad Tabari A, Soori H, Maracy M. Evaluation of incidence and main risk factors of major congenital anomalies in hospitals affiliated with Isfahan University of Medical Sciences during 1395. Iran J Epidemiol 2020;16(1):48-56. [Persian].

[65] Masoodpoor N, Arab-Baniasad F, Jafari A. Prevalence and pattern of congenital malformations in newborn in Rafsanjan, Iran (2007-2008). J Gorgan Univ Med Sci 2013;15(3):114-117. [Persian].

[66] Parandavar A. Investigating the Number of Congenital Anomalies in Babies Born at Imam Khomeini Hospital in Islamabad Gharb City from 2017-2022. Kermanshah: Faculty of Agricultural Payam Noor University; 2023.

[67] Ghahramani M, Moshki M, Ebadi A. A survey of causes and prevalence of congenital anomalies in live born neonates in Gonabad 22 Bahman hospital (1373-1380). Intern Med Today 2002;8(1):1-6. [Persian].

[68] Khatami F, Mamuri GA. Survey of congenital major malformation in 10,000 newborns. Iran J Pediatr 2005;15(4):315-320. [Persian].

[69] Ahmadzadeh A, Safikhani Z, Abdulahi M, Ahmadzadeh A (2008). Congenital malformations among live births at Arvand hospital, Ahwaz, Iran-a prospective study. Pak J Med Sci.

[70] Rahim F, Ebadi A, Saki G, Ramezani A (2008). Prevalence of congenital heart disease in Iran: a clinical study. J Med Sci.

[71] Khoshhal-Rahdar F, Saadati H, Mohammadian M, Hafar-Rangchi M, Mohazzab-Torabi S, Khabazkhoob M. The prevalence of congenital malformations in Dezful-2012. Genetics in the Third Millennium 2014;12(3):3622-3631. [Persian].

[72] Mohsenzadeh A, Saket S, Ahmadipour S, Baharvand B. Prevalence and types of congenital heart disease in babies born in the city of Khorramabad (2007-2011). Yafteh 2014;15(5):23-29. [Persian].

[73] Mohammadzadeh I, Sorkhi H, Alizadeh-Navaei R (2013). Prevalence of external congenital malformations in neonates born in Mehregan hospital, north of Iran. Iran J Neonatol.

[74] Movafagh A, Pear Zadeh Z, Hajiseyed Javadi M (2008). Occurrence of congenital anomalies and genetic diseases in a population of Ghazvin province, Iran: a study of 33380 cases. Pak J Med Sci.

[75] Farhud DD, Walizadeh GR, Kamali MS (1986). Congenital malformations and genetic diseases in Iranian infants. Hum Genet.

[76] Vakilian K, Hajian S, Sadeghian A (2013). Frequency of congenital structural anomalies in newborns of Shahroud, Iran. Zahedan J Res Med Sci.

[77] Hosseini S, Nikravesh A, Hashemi Z, Rakhshi N (2014). Race of apparent abnormalities in neonates born in Amir-Almomenin hospital of Sistan. J North Khorasan Uni Med Sci.

[78] Asemi-Rad A, Heidari Z, Mahmoudzadeh-Sagheb H (2023). Prevalence of congenital anomalies and related factors in live births in Zahedan, southeast of Iran: a cross-sectional study. Int J Reprod Biomed.

[79] Rahnama F, Hashemian M, Akbarzadeh R, Akabari A. The incidence of apparent congenital anomalies in neonates in Mobini Maternity Hospital in Sabzevar Iran in 2005-6. J Sabzevar Univ Med Sci 2009;15(4):231-236. [Persian].

[80] Amini Nasab Z, Aminshokravi F, Moodi M, Eghbali B, Fatemimogadam F. Demographical condition of neonates with congenital abnormalities under Birjand city health centers during 2007-2012. J Birjand Univ Med Sci 2014;21(1):96-103. [Persian].

[81] Bordbar A, Kashaki M, Vafapour M, Sepehri A (2023). Determining the incidence of heart malformations in neonates: a novel and clinically approved solution. Front Pediatr.

[82] Tootoonchi P (2003). Easily identifiable congenital anomalies: prevalence and risk factors. Acta Med Iran.

[83] Hematyar M, Khajouie P. Prevalence of congenital anomalies in 1000 live births in Javaheri hospital, Tehran, 2004. Medical Sciences Journal of Islamic Azad University 2005;15(2):75-78. [Persian].

[84] Delshad S, Khalegnejad Tabar A, Samae H (2009). The incidence of selected congenital malformations during a two-year period in Tehran, Iran. Trop Doct.

[85] Farhangniya M, Dortaj Rabori E, Mozafari Kermani R (2013). Comparison of congenital abnormalities of infants conceived by assisted reproductive techniques versus infants with natural conception in Tehran. Int J Fertil Steril.

[86] Abdi-Rad I, Khoshkalam M, Farrokh-Islamlou HR (2008). The prevalence at birth of overt congenital anomalies in Urmia, northwestern Iran. Arch Iran Med.

[87] Akhavan Karbasi S, Golestan M, Fallah R, Mirnaseri F, Barkhordari K, Sadr Bafghi M (2009). Prevalence of congenital malformations in Yazd (Iran). Acta Med Iran.

[88] Tayebi N, Yazdani K, Naghshin N (2010). The prevalence of congenital malformations and its correlation with consanguineous marriages. Oman Med J.

[89] Taheri M, Dehghani A, Noorishadkam M, Tabatabaei SM (2015). Population attributable danger of hereditary heart breaks Risk factors among newborns in Yazd, Iran. J Med Life.

[90] Rafati S. Examining the Frequency and Types of Congenital Anomalies of Newborns Born in Hospitals in Yazd Province During the Years 2016-2022. Tehran: Faculty of Medicine, Shahed University; 2023.

[91] Safaei Nezhad A, Mahmoudi M, Kharaghani R (2018). The prevalence of birth defects and related factors in Zanjan city (northwest, Iran) during 2015-2016. J Adv Med Biomed Res.

[92] Radvar M, Zolfi A, Fakour Z, Farhadi E (2022). Epidemiology of clinical findings and outcome in neonates with congenital heart disease. Health Sci Monit.

[93] Amiri Simkouii F, Jamshidi M, Behjati Ardakani M, Toosi F, Alipour MR, Namayandeh SM (2021). Comparative study of pulse oximetry, physical examination and echocardiography results in the diagnosis of congenital heart defects in neonates in the first 24 hours of life. J Shahid Sadoughi Univ Med Sci.

[94] Reller MD, Strickland MJ, Riehle-Colarusso T, Mahle WT, Correa A (2008). Prevalence of congenital heart defects in metropolitan Atlanta, 1998-2005. J Pediatr.

[95] Jullien S (2021). Newborn pulse oximetry screening for critical congenital heart defects. BMC Pediatr.

[96] Mocumbi AO, Lameira E, Yaksh A, Paul L, Ferreira MB, Sidi D (2011). Challenges on the management of congenital heart disease in developing countries. Int J Cardiol.

[97] Li JJ, Liu Y, Xie SY (2019). Newborn screening for congenital heart disease using echocardiography and follow-up at high altitude in China. Int J Cardiol.

[98] Sokal R, Tata LJ, Fleming KM (2014). Sex prevalence of major congenital anomalies in the United Kingdom: a national population-based study and international comparison meta-analysis. Birth Defects Res A Clin Mol Teratol.

[99] Jenkins KJ, Correa A, Feinstein JA (2007). Noninherited risk factors and congenital cardiovascular defects: current knowledge: a scientific statement from the American Heart Association Council on Cardiovascular Disease in the Young: endorsed by the American Academy of Pediatrics. Circulation.

[100] Edler I, Lindström K (2004). The history of echocardiography. Ultrasound Med Biol.

[101] Lunn D, Barrett J, Sweeting M, Thompson S (2013). Fully Bayesian hierarchical modelling in two stages, with application to meta-analysis. J R Stat Soc Ser C Appl Stat.

[102] Tikkanen J, Heinonen OP (1990). Risk factors for cardiovascular malformations in Finland. Eur J Epidemiol.

