# Supplementary material for: Spatial Distribution and Birth Prevalence of Congenital Heart Disease in Iran: A Systematic Review and Hierarchical Bayesian Meta-analysis
Source: Int J Health Policy Manag. 2024 May 7;13:7931. doi: 10.34172/ijhpm.2024.7931 (PMC11270618; doi:10.34172/ijhpm.2024.7931)
Supplement: Supplementary file 1 — Search Strategy in the Mentioned Databases. [file ijhpm-13-7931-s001.pdf]

Article title: Spatial Distribution and Birth Prevalence of Congenital Heart Disease in Iran: A Systematic Review and Hierarchical Bayesian Meta-analysis

Journal name: International Journal of Health Policy and Management (IJHPM)

Authors' information: Roghaye Farhadi Hassankiadeh[1], Annette Dobson[2], Somayeh Rahimi[3], Abdollah Jalilian[4], Volker J Schmid[5], Behzad Mahaki[1]*

[1]Department of Biostatistics, School of Health, Kermanshah University of Medical Sciences, Kermanshah, Iran.

[2]School of Public Health, University of Queensland, Brisbane, QLD, Australia.

[3]Department of Clinical Biochemistry, Kermanshah University of Medical Sciences, Kermanshah, Iran.

[4]Department of Statistics, Razi University, Kermanshah, Iran.

[5]Department of Statistics, Ludwig-Maximilians-University, Munich, Germany.

***Correspondence to:** Behzad Mahaki; Email: behzad.mahaki@gmail.com

Citation: Farhadi Hassankiadeh R, Dobson A, Rahimi S, Jalilian A, Schmid VJ, Mahaki B. Spatial distribution and birth prevalence of congenital heart disease in Iran: a systematic review and hierarchical Bayesian meta-analysis. Int J Health Policy Manag. 2024;13:7931. doi:10.34172/ijhpm.2024.7931

Supplementary file 1. Search Strategy in the Mentioned Databases

4 October 2023:

Table 1. search strategy in web of science

**Number**	**Strategy**	**# of Hits**
**Diseases**		
**1**	ALL=(("Congenital heart" OR "Congenital heart disease" OR "congenital heart defect" OR "congenital heart malformation" OR "congenital heart anomaly" OR "congenital heart anomalies" OR "congenital cardiac disease" OR "congenital cardiac defect" OR "congenital cardiac malformation" OR "congenital cardiac anomaly" OR "congenital cardiac anomalies" OR "congenital cardiovascular disease" OR "cardiovascular malformation" OR "cardiovascular defect" OR "cardiovascular anomalies" OR "cardiovascular anomaly" OR "birth defect" OR "congenital malformation" OR "congenital anomalies" OR "congenital anomaly" OR "congenital disorders" OR "Congenital Abnormalities" OR "Congenital Abnormality" OR "malformation in neonate" OR "malformations in neonates" OR "abnormality in neonate" OR "abnormalities in neonates" OR "congenital defect"))	95,607
**Outcome**		
**2**	ALL=(prevalence OR incidence OR frequency OR epidemiology)	4,276,234
**Disease + Outcome**		
**3**	#1 AND #2	17,529
**Country**		
**4**	ALL=(Iran OR Iranian)	641,073
**5**	#3 AND #4	297

Table 2. search strategy in PubMed		
**Number**	**Strategy**	**# of Hits**
**Diseases**		
**1**	("Congenital heart"[All Fields] OR "Congenital heart disease"[All Fields] OR "congenital heart defect"[All Fields] OR "congenital heart malformation"[All Fields] OR "congenital heart anomaly"[All Fields] OR "congenital heart anomalies"[All Fields] OR "congenital cardiac disease"[All Fields] OR "congenital cardiac defect"[All Fields] OR "congenital cardiac malformation"[All Fields] OR "congenital cardiac anomaly"[All Fields] OR "congenital cardiac anomalies"[All Fields] OR "congenital cardiovascular disease"[All Fields] OR "cardiovascular malformation"[All Fields] OR "cardiovascular defect"[All Fields] OR "cardiovascular anomalies"[All Fields] OR "cardiovascular anomaly"[All Fields] OR "birth defect"[All Fields] OR "congenital malformation"[All Fields] OR "congenital anomalies"[All Fields] OR "congenital anomaly"[All Fields] OR "congenital disorders"[All Fields] OR "Congenital Abnormalities"[All Fields] OR "Congenital Abnormality"[All Fields] OR (("abnormalities"[MeSH Subheading] OR "abnormalities"[All Fields] OR "malformations"[All Fields] OR "Congenital Abnormalities"[MeSH Terms] OR ("congenital"[All Fields] AND "abnormalities"[All Fields]) OR "Congenital Abnormalities"[All Fields] OR "malformation"[All Fields] OR "malformational"[All Fields] OR "malformative"[All Fields] OR "malformed"[All Fields]) AND ("infant, newborn"[MeSH Terms] OR ("infant"[All Fields] AND "newborn"[All Fields]) OR "newborn infant"[All Fields] OR "neonatal"[All Fields] OR "neonate"[All Fields] OR "neonates"[All Fields] OR "neonatality"[All Fields] OR "neonatals"[All Fields] OR "neonate s"[All Fields])) OR "malformations in neonates"[All Fields] OR (("abnormal"[All Fields] OR "abnormalities"[MeSH Subheading] OR "abnormalities"[All Fields] OR "Congenital Abnormalities"[MeSH Terms] OR ("congenital"[All Fields] AND "abnormalities"[All Fields]) OR "Congenital Abnormalities"[All Fields] OR "abnormality"[All Fields] OR "abnormally"[All Fields] OR "abnormals"[All Fields] OR "abnormities"[All Fields] OR "abnormity"[All Fields]) AND ("infant, newborn"[MeSH Terms] OR ("infant"[All Fields] AND "newborn"[All Fields]) OR "newborn infant"[All Fields] OR "neonatal"[All Fields] OR "neonate"[All Fields] OR "neonates"[All Fields] OR "neonatality"[All Fields] OR "neonatals"[All Fields] OR "neonate s"[All Fields])) OR (("abnormal"[All Fields] OR "abnormalities"[MeSH Subheading] OR "abnormalities"[All Fields] OR "Congenital Abnormalities"[MeSH Terms] OR ("congenital"[All Fields] AND "abnormalities"[All Fields]) OR "Congenital Abnormalities"[All Fields] OR "abnormality"[All	288,267

	Fields] OR "abnormally"[All Fields] OR "abnormals"[All Fields] OR "abnormities"[All Fields] OR "abnormity"[All Fields]) AND ("infant, newborn"[MeSH Terms] OR ("infant"[All Fields] AND "newborn"[All Fields]) OR "newborn infant"[All Fields] OR "neonatal"[All Fields] OR "neonate"[All Fields] OR "neonates"[All Fields] OR "neonatality"[All Fields] OR "neonatals"[All Fields] OR "neonate s"[All Fields])) OR "congenital defect"[All Fields])	
**Outcome**		
**2**	" ("epidemiology"[MeSH Subheading] OR "epidemiology"[All Fields] OR "prevalence"[All Fields] OR "prevalence"[MeSH Terms] OR "prevalance"[All Fields] OR "prevalences"[All Fields] OR "prevalence s"[All Fields] OR "prevalent"[All Fields] OR "prevalently"[All Fields] OR "prevalents"[All Fields] OR "epidemiology"[MeSH Subheading] OR "epidemiology"[All Fields] OR "incidence"[All Fields] OR "incidence"[MeSH Terms] OR "incidences"[All Fields] OR "incident"[All Fields] OR "incidents"[All Fields] OR "epidemiology"[MeSH Subheading] OR "epidemiology"[All Fields] OR "frequency"[All Fields] OR "epidemiology"[MeSH Terms] OR "frequence"[All Fields] OR "frequences"[All Fields] OR "frequencies"[All Fields] OR "epidemiologies"[All Fields] OR "epidemiology"[MeSH Subheading] OR "epidemiology"[All Fields] OR "epidemiology"[MeSH Terms] OR "epidemiology s"[All Fields])	5,109,468
**Disease + Outcome**		
**3**	#1 AND #2	68,396
**Country**		
**4**	("iran"[MeSH Terms] OR "iran"[All Fields] OR "iranian people"[Supplementary Concept] OR "iranian people"[All Fields] OR "iranians"[All Fields] OR "iranian"[All Fields] OR "iranian s"[All Fields])	267,523
**5**	#3 AND #4	556

Table 3. search strategy in Scopus		
**Number**	**Strategy**	**# of Hits**
**Diseases**		
**1**	TITLE-ABS-KEY ("Congenital heart" OR "Congenital heart disease" OR "congenital heart defect" OR "congenital heart malformation" OR "congenital heart anomaly" OR "congenital heart anomalies" OR "congenital cardiac disease" OR "congenital cardiac defect" OR "congenital cardiac malformation" OR "congenital cardiac anomaly" OR "congenital cardiac anomalies" OR "congenital cardiovascular disease" OR "cardiovascular malformation" OR "cardiovascular defect" OR "cardiovascular anomalies" OR "cardiovascular anomaly" OR "birth defect" OR "congenital malformation" OR "congenital anomalies" OR "congenital anomaly" OR "congenital disorders" OR "Congenital Abnormalities" OR "Congenital Abnormality" OR "malformation in neonate" OR "malformations in neonates" OR "abnormality in neonate" OR "abnormalities in neonates" OR "congenital defect")	375,367
**Outcome**		
**2**	TITLE-ABS-KEY (prevalence OR incidence OR frequency OR epidemiology)	6,950,314
**Disease + Outcome**		
**3**	#1 AND #2	52,178
**Country**		
**4**	TITLE-ABS-KEY (iran OR iranian)	211,778
**5**	#3 AND #4	351

Table 4. search strategy in Science Direct		
**Number**	**Strategy**	**# of Hits**
**Diseases**		
**1**	Title, abstract, keywords: ("Congenital heart" OR "congenital cardiac" OR "birth defect" OR "congenital malformation" OR "congenital anomalies" OR "congenital defect")	933
**2**	Title, abstract, keywords: (Congenital heart OR congenital cardiac OR birth defect OR congenital malformation OR congenital anomalies OR congenital defect)	1,720
**Outcome**		
**3**	Title, abstract, keywords: (prevalence OR incidence)	19,782
**4**	Title, abstract, keywords: (frequency OR epidemiology)	31,754
**Disease + Outcome**		
**5**	#1 AND #3	644
**6**	#2 AND #3	1,134
**7**	#2 AND #4	1,011
**Country**		
**8**	Title, abstract, keywords: (iran)	6,128
**9**	#1 AND #3 AND #7	110
**10**	#2 AND #3 AND #7	189
**11**	#2 AND #4 AND #7	160
**12**	9 + 10+ 11= 459 – duplicates (248) *	211

*In Science Direct, we encountered a limit on the number of Boolean operators (up to 8 per field). Consequently, we had to divide the keywords into 3 parts.

Table 5. search strategy in Iranian Database			
**Databases**	Set	Search terms	results
**SID**	1	congenital(title) prevalence(filter) in Persian	608
	2	congenital(title) prevalence(filter)	536
**Magiran**	1	congenital prevalence in Persian	392
	2	congenital prevalence	576
**IranDoc**	1	congenital prevalence in Persian	271
