# Supplementary material for: Spatial Distribution and Birth Prevalence of Congenital Heart Disease in Iran: A Systematic Review and Hierarchical Bayesian Meta-analysis
Source: Int J Health Policy Manag. 2024 May 7;13:7931. doi: 10.34172/ijhpm.2024.7931 (PMC11270618; doi:10.34172/ijhpm.2024.7931)
Supplement: Supplementary file 2 — Quality Assessment (ARHQ Methodology) of Checklist for Cross-sectional/Prevalence Study. [file ijhpm-13-7931-s002.pdf]

Article title: Spatial Distribution and Birth Prevalence of Congenital Heart Disease in Iran: A Systematic Review and Hierarchical Bayesian Meta-analysis

Journal name: International Journal of Health Policy and Management (IJHPM)

Authors' information: Roghaye Farhadi Hassankiadeh[1], Annette Dobson[2], Somayeh Rahimi[3], Abdollah Jalilian[4], Volker J Schmid[5], Behzad Mahaki[1]*

[1]Department of Biostatistics, School of Health, Kermanshah University of Medical Sciences, Kermanshah, Iran.

[2]School of Public Health, University of Queensland, Brisbane, QLD, Australia.

[3]Department of Clinical Biochemistry, Kermanshah University of Medical Sciences, Kermanshah, Iran.

[4]Department of Statistics, Razi University, Kermanshah, Iran.

[5]Department of Statistics, Ludwig-Maximilians-University, Munich, Germany.

***Correspondence to:** Behzad Mahaki; Email: behzad.mahaki@gmail.com

Citation: Farhadi Hassankiadeh R, Dobson A, Rahimi S, Jalilian A, Schmid VJ, Mahaki B. Spatial distribution and birth prevalence of congenital heart disease in Iran: a systematic review and hierarchical Bayesian meta-analysis. Int J Health Policy Manag. 2024;13:7931. doi:10.34172/ijhpm.2024.7931

Supplementary file 2. Quality Assessment (ARHQ Methodology) of Checklist for Cross-sectional/Prevalence Study

ID	First author	I1	I2	I3	I4	I5	I6	I7	I8	I9	I10	I11	Total score
1	Naderi, S	Yes	Yes	Yes	Yes	Yes	Unclear	Unclear	Unclear	Yes	Yes	No	7
2	Farhud, D	Yes	Yes	Yes	Unclear	No	Yes	Unclear	Yes	Yes	Yes	No	7
3	Shahmohammadi, F	Yes	Yes	Yes	Unclear	Unclear	Yes	Yes	Yes	Yes	Yes	No	8
4	Zamani, A	Yes	Yes	Yes	Unclear	Yes	Yes	No	Yes	Yes	Yes	No	8
5	Movahedian, A	Yes	Yes	Yes	Unclear	Yes	Yes	Yes	Yes	Yes	Yes	No	9
6	Ghahramani, M	Yes	Yes	Yes	Yes	Yes	No	Yes	Unclear	Yes	Yes	Unclear	8
7	Toutounchi, P	Yes	Yes	Yes	Yes	Yes	No	Yes	Unclear	Yes	Yes	Unclear	8
8	Akbari, M	Yes	Yes	Yes	No	Yes	Yes	No	Yes	Yes	Yes	No	8
9	Ghorbani, M	Yes	Yes	Yes	No	Yes	Yes	No	Yes	Yes	Yes	No	8
10	Khatami, F	Yes	Yes	Yes	No	Yes	Yes	No	Yes	Yes	Yes	No	8

ID	First author	I1	I2	I3	I4	I5	I6	I7	I8	I9	I10	I11	Total score
11	Pouladfar, Gh	Yes	Yes	Yes	No	Yes	Yes	No	Yes	Yes	Yes	No	8
12	JGolalipour,M.J	Yes	Yes	Yes	No	Yes	Yes	No	Yes	Yes	Yes	No	8
13	Hematyar, M	Yes	Yes	Yes	Yes	Yes	Yes	No	Yes	Yes	Yes	No	9
14	Shajari,H	Yes	Yes	Yes	No	Yes	Yes	No	Yes	Yes	Yes	No	8
15	Dastgiri, S	Yes	Yes	Yes	Unclear	Yes	Yes	Yes	Yes	Yes	Yes	Unclear	9
16	Mosayebi, Z	Yes	Yes	Yes	No	Yes	Yes	Yes	Yes	Yes	Yes	Unclear	9
17	Movafagh, A	Yes	Yes	Yes	No	Unclear	Yes	No	Yes	Yes	Yes	Yes	8
18	Ahmadzadeh, A	Yes	Yes	Yes	No	Unclear	Yes	No	Yes	Yes	Yes	No	7
19	Rahim, F	Yes	Yes	Yes	Unclear	Yes	Yes	Yes	Yes	Yes	Yes	Unclear	9
20	Abdi-Rad, I	Yes	Yes	Yes	No	Unclear	Yes	No	Yes	Yes	Yes	No	7
21	Sereshti, M	Yes	Yes	Yes	No	Unclear	Yes	No	Yes	Yes	Yes	No	7
22	Aliakbarzadeh,R	Yes	Yes	Yes	No	Unclear	Yes	No	Yes	Yes	Yes	No	7
23	Delshad, S	Yes	Yes	Yes	No	Unclear	Yes	No	Yes	Yes	Yes	No	7
24	Akhavan Karbasi, S	Yes	Yes	Yes	Yes	Yes	Yes	No	Yes	Yes	Yes	No	9
25	Tayebi, N	Yes	Yes	Yes	Yes	Yes	Yes	No	Yes	Unclear	Yes	No	8
26	Mirzarahimi, M	Yes	Yes	Yes	No	Yes	Yes	No	Yes	Unclear	Yes	No	7
27	Dastgiri, S	Yes	Yes	Yes	Unclear	Yes	Yes	Yes	Yes	Unclear	Yes	No	8
28	Nikyar, B	Yes	Yes	Yes	No	Yes	Yes	No	Yes	Unclear	Yes	No	7
29	Samadirad, B	Yes	Yes	Yes	Unclear	Yes	Yes	Yes	Yes	Unclear	Yes	No	8
30	Nazemi Gheshmi,A	Yes	Yes	Yes	No	Yes	Yes	No	Yes	No	Yes	No	7
31	Farhangniya, M	Yes	Yes	Yes	No	Yes	Yes	No	Yes	Unclear	Yes	No	7
32	Alijahan, R	Yes	Yes	Yes	Unclear	Yes	Yes	Yes	Yes	Unclear	Yes	No	8
33	Golalipour, M.J	Yes	Yes	Yes	Unclear	Yes	Yes	Yes	Yes	Unclear	Yes	No	8
34	Masoodpoor, N	Yes	Yes	Yes	Unclear	Yes	Yes	Yes	Yes	Unclear	Yes	No	8

ID	First author	I1	I2	I3	I4	I5	I6	I7	I8	I9	I10	I11	Total score
35	Vakilian, K	Yes	Yes	Yes	Unclear	Yes	Yes	Yes	Yes	Unclear	Yes	No	8
36	Mohsenzadeh, A	Yes	Yes	Yes	Unclear	Yes	Yes	Yes	Yes	Unclear	Yes	No	8
37	Mashhadi Abdolahi, H	Yes	Yes	Yes	Unclear	Yes	Yes	Yes	Yes	Unclear	Yes	No	8
38	Nikyar, B	Yes	Yes	Yes	No	Yes	Yes	Yes	Yes	Unclear	Yes	No	8
39	Khoshhal-Rahdar, F	Yes	Yes	Yes	No	Yes	Yes	Yes	Yes	Unclear	Yes	No	8
40	Hosseini, S	Yes	Yes	Yes	No	Yes	Yes	Yes	Yes	Unclear	Yes	No	8
41	Amini Nasab, Z	Yes	Yes	Yes	No	Yes	Yes	Yes	Yes	Unclear	Yes	No	8
42	Bagheri, M.M	Yes	Yes	Yes	No	Yes	Yes	Yes	Yes	Unclear	Yes	No	8
43	Jalali, S.Z	Yes	Yes	Yes	No	Yes	Yes	Yes	Yes	Unclear	Yes	No	8
44	Taheri, M	Yes	Yes	Yes	No	Yes	Yes	Yes	Yes	Unclear	Yes	No	8
45	Sayehmiri, K	Yes	Yes	Yes	No	Yes	Yes	Yes	Yes	Unclear	Yes	No	8
46	Stone, D. H.	Yes	Yes	Yes	No	Yes	Yes	Yes	Yes	Unclear	Yes	Yes	9
47	Rostamizadeh, L	Yes	Yes	Yes	Yes	Yes	Yes	Yes	Yes	Unclear	Yes	Yes	10
48	Movahedian, A.H	Yes	Yes	Yes	No	Yes	Yes	Yes	Yes	Unclear	Yes	No	8
49	Mirfazeli, A	Yes	Yes	Yes	No	Yes	Yes	Yes	Yes	Unclear	Yes	Yes	9
50	Safaei Nezhad, A	Yes	Yes	Yes	No	Yes	Yes	Yes	Yes	Unclear	Yes	Yes	9
51	Davari, H.A	Yes	Yes	Yes	No	Yes	Yes	Yes	Yes	Unclear	Yes	Unclear	8
52	Saberi, M	Yes	Yes	Yes	No	Yes	Yes	Yes	Yes	Unclear	Yes	Unclear	8
53	Molapour, H	Yes	Yes	Yes	No	Yes	Yes	Yes	Yes	Unclear	Yes	Yes	9
54	Tarighat, F	Yes	Yes	Yes	No	Yes	Yes	Yes	Yes	Unclear	Yes	Yes	9
55	Vafaei, H	Yes	Yes	Yes	Yes	Yes	Yes	Yes	Yes	Unclear	Yes	No	9
56	Mohammadzadeh, I	Yes	Yes	Yes	No	Yes	Yes	Yes	Yes	Unclear	Yes	Unclear	4
57	Amiri Simkouii, F	Yes	Yes	Yes	No	Yes	Yes	Yes	Yes	Unclear	Yes	No	8
58	Radvar, M	Yes	Yes	Yes	No	Yes	Yes	Yes	Yes	Unclear	Yes	No	8

ID	First author	I1	I2	I3	I4	I5	I6	I7	I8	I9	I10	I11	Total score
59	Heidarzadeh, M	Yes	Yes	Yes	No	Yes	Yes	Yes	Yes	Unclear	Yes	Yes	9
60	Rafati, Sh	Yes	Yes	Yes	No	Yes	Yes	No	Yes	Unclear	Yes	Yes	8
61	Arandavar, A	Yes	Yes	Yes	No	Yes	Yes	No	Yes	Unclear	Yes	No	7
62	Asemi-Rad, A	Yes	Yes	Yes	No	Yes	Yes	No	Yes	Unclear	Yes	No	7

Notes: Yes = Yes; 0 = No; 0 = Unclear; Agency for Healthcare Research and Quality (AHRQ) methodology checklist, which included 11 items: (I1) Define the source of information; (I2) List inclusion and exclusion criteria for exposed and unexposed subjects or provide a reference to previous publications that describe these criteria; (I3) Indicate time period used for identifying patients; (I4) Indicate whether or not subjects were consecutive if not population-based; (I5) Indicate if evaluators of subjective components of were masked to other aspects of the status of the participants; (I6) Describe any assessments undertaken for quality control purposes; (I7) Explain any patient exclusions from analysis; (I8) Describe how confounding was assessed and/or controlled; (I9) If applicable, explain how missing data were handled in the analysis; (I10) Summarize patient response rates and completeness of data collection; (I11) Clarify what follow-up, if any, was expected and the percentage of patients for which incomplete data or follow-up was obtained.
