# Supplementary material for: Spatial Distribution and Birth Prevalence of Congenital Heart Disease in Iran: A Systematic Review and Hierarchical Bayesian Meta-analysis
Source: Int J Health Policy Manag. 2024 May 7;13:7931. doi: 10.34172/ijhpm.2024.7931 (PMC11270618; doi:10.34172/ijhpm.2024.7931)
Supplement: Supplementary file 3 — Bayesian Hierarchical Pooling of CHD Birth Prevalence. [file ijhpm-13-7931-s003.pdf]

Article title: Spatial Distribution and Birth Prevalence of Congenital Heart Disease in Iran: A Systematic Review and Hierarchical Bayesian Meta-analysis

Journal name: International Journal of Health Policy and Management (IJHPM)

Authors' information: Roghaye Farhadi Hassankiadeh[1], Annette Dobson[2], Somayeh Rahimi[3], Abdollah Jalilian[4], Volker J Schmid[5], Behzad Mahaki[1]*

[1]Department of Biostatistics, School of Health, Kermanshah University of Medical Sciences, Kermanshah, Iran.

[2]School of Public Health, University of Queensland, Brisbane, QLD, Australia.

[3]Department of Clinical Biochemistry, Kermanshah University of Medical Sciences, Kermanshah, Iran.

[4]Department of Statistics, Razi University, Kermanshah, Iran.

[5]Department of Statistics, Ludwig-Maximilians-University, Munich, Germany.

***Correspondence to:** Behzad Mahaki; Email: behzad.mahaki@gmail.com

Citation: Farhadi Hassankiadeh R, Dobson A, Rahimi S, Jalilian A, Schmid VJ, Mahaki B. Spatial distribution and birth prevalence of congenital heart disease in Iran: a systematic review and hierarchical Bayesian meta-analysis. Int J Health Policy Manag. 2024;13:7931. doi:10.34172/ijhpm.2024.7931

Supplementary file 3. Bayesian Hierarchical Pooling of CHD Birth Prevalence

The posterior density is denoted by p (μ,τ|y,σ). This is the conditional distribution of μ and τ and given the values of y and σ[1] .

Let Y_i denote the number of people with CHD in the i-th paper, N_i the corresponding total sample size, and p_i the underlying prevalence. First, Y_i follows a $Binomial$ distribution, so we have $Y_i|p_i \sim Binomial(N_i, p_i)$. Next, we assume p_i is a random variable with a probability density function. Therefore, the logit transformation of p_i has a Normal distribution. We assume

$$logit(\, p_i) = \log\left(\frac{p_i}{1 - p_i}\right) = u_i \; and \; u_i \sim Normal(\mu, \tau^2)$$

For the mean μ of the normal distribution of logarithm of odds a weakly informative normal prior distribution (with mean= 0 and variance = 100), and for the variance τ^2 a slightly more informative half-normal prior distribution (with variance=0.5) [1-3]

Finally, We converted these estimates (logit[1]) back to the prevalence with the following formula:

P= [exp(log odds)/(exp(log odds) + 1)]

1. Röver C. An introduction to meta-analysis using the bayesmeta package. https://cran.r-project.org/web/packages/bayesmeta/vignettes/bayesmeta.html.

2. Röver C, Bender R, Dias S, et al. On weakly informative prior distributions for the heterogeneity parameter in Bayesian random-effects meta-analysis. *Res Synth Methods.* 2021;12(4):448-474. doi:10.1002/jrsm.1475

3. Mohammadi FS, Aslani S, Mostafaei S, Jamshidi A, Riahi P, Mahmoudi M. Are genetic variations in IL-21-IL-23R-IL-17A cytokine axis involved in a pathogenic pathway of rheumatoid arthritis? Bayesian hierarchical meta-analysis. *J Cell Physiol.* 2019;234(10):17159-17171. doi:10.1002/jcp.28495

[1] log odds
