# Supplementary material for: Spatial Distribution and Birth Prevalence of Congenital Heart Disease in Iran: A Systematic Review and Hierarchical Bayesian Meta-analysis
Source: Int J Health Policy Manag. 2024 May 7;13:7931. doi: 10.34172/ijhpm.2024.7931 (PMC11270618; doi:10.34172/ijhpm.2024.7931)
Supplement: Supplementary file 4 — contains Tables S1-S3. [file ijhpm-13-7931-s004.pdf]

Article title: Spatial Distribution and Birth Prevalence of Congenital Heart Disease in Iran: A Systematic Review and Hierarchical Bayesian Meta-analysis

Journal name: International Journal of Health Policy and Management (IJHPM)

Authors' information: Roghaye Farhadi Hassankiadeh[1], Annette Dobson[2], Somayeh Rahimi[3], Abdollah Jalilian[4], Volker J Schmid[5], Behzad Mahaki[1]*

[1]Department of Biostatistics, School of Health, Kermanshah University of Medical Sciences, Kermanshah, Iran.

[2]School of Public Health, University of Queensland, Brisbane, QLD, Australia.

[3]Department of Clinical Biochemistry, Kermanshah University of Medical Sciences, Kermanshah, Iran.

[4]Department of Statistics, Razi University, Kermanshah, Iran.

[5]Department of Statistics, Ludwig-Maximilians-University, Munich, Germany.

***Correspondence to:** Behzad Mahaki; Email: behzad.mahaki@gmail.com

Citation: Farhadi Hassankiadeh R, Dobson A, Rahimi S, Jalilian A, Schmid VJ, Mahaki B. Spatial distribution and birth prevalence of congenital heart disease in Iran: a systematic review and hierarchical Bayesian meta-analysis. Int J Health Policy Manag. 2024;13:7931. doi:10.34172/ijhpm.2024.7931

Supplementary file 4

Table 1

Summary of studies included in the review

ID	First author	Year of publication	Total sample size	Event of CHD	Province	Study period	Age	Male	Female
**1**	Naderi, S	1979	9623	11	Fars	1972-1975	0-1	-	-
**2**	Farhud, D	1986	13037	25	Tehran	1969 -1977	0-1	12	13
**3**	Shahmohammadi, F	1997	2510	0	Markazi	1997- 1998	0-1	-	-
**4**	Zamani, A	2000	4073	8	Tehran	1999-2000	0-1	-	-
**5**	Movahedian, A	2001	2840	46	Isfahan	1996-2000	0-1	26	25
**6**	Ghahramani, M	2002	12319	1	Razavi Khorasan	1994 - 2001	0-1	-	-
**7**	Toutounchi, P	2003	2291	2	Tehran	1999 - 2000	0-1	-	-
**8**	Akbari, M	2003	6424	11	Tehran	2000 -2001	0-1	-	-
**9**	Ghorbani, M	2004	12853	1	Golestan	2000-2001	0-1	-	-
**10**	Khatami, F	2005	10450	11	Razavi Khorasan	1991-1993	0-1	-	-
**11**	Pouladfar, Gh	2005	722	0	Bushehr	2002-2003	0-1	-	-
**12**	JGolalipour,M.J	2005	10000	0	Golestan	1997-1999	0-1	-	-
**13**	Hematyar, M	2005	1000	2	Tehran	2003 - 2018	0-1	-	-
**14**	Shajari,H	2006	3840	0	Tehran	2002-2004	0-1	-	-

ID	First author	Year of publication	Total sample size	Event of CHD	Province	Study period	Age	Male	Female
**15**	Dastgiri, S	2007	95119	129	East Azerbaijan	2000 -2004	0-1	-	-
**16**	Mosayebi, Z	2007	3529	16	Isfahan	2001 -2002	0-1	-	-
**17**	Movafagh, A	2008	33380	202	Ghazvin	2000- 2004	0-8 years	90	112
**18**	Ahmadzadeh, A	2008	4660	3	Khuzestan	2003- 2006	0-1	-	-
**19**	Rahim, F	2008	217250	1544	Khuzestan	1998 - 2007	0-10 years	-	79
**20**	Abdi-Rad, I	2008	14121	0	West Azerbaijan	2001- 2005	0-1	-	-
**21**	Sereshti, M	2008	2854	0	Chaharmahal and Bakhtiari	2005-2006	0-1	-	-
**22**	Aliakbarzadeh,R	2009	7786	13	South Khorasan	2005 -2006	0-1	9	4
**23**	Delshad, S	2009	61112	12	Tehran	2005 -2007	0-1	4	8
**24**	Akhavan Karbasi, S	2009	4800	9	Yazd	2003 - 2004	0-1	-	-
**25**	Tayebi, N	2010	1195	4	Yazd	2008-2008	0-1	-	-
**26**	Mirzarahimi, M	2011	2928	47	Ardabil	2006 - 2007	0-1	-	-
**27**	Dastgiri, S	2011	185650	451	East Azerbaijan	2000 - 2009	0-1	-	-
**28**	Nikyar, B	2011	11739	97	Golestan	2007- 2008	0-1	56	41
**29**	Samadirad, B	2012	22524	12	East Azerbaijan	2010-2011	0-1	-	-
**30**	Nazemi Gheshmi,A	2012	7007	0	Hormozgan	2007-2008	0-1	-	-
**31**	Farhangniya, M	2013	978	12	Tehran	2008 -2010	0-1	-	-
**32**	Alijahan, R	2013	6868	0	Ardabil	2010-2011	0-1	-	-
**33**	Golalipour, M.J	2013	6204	32	Golestan	2007-2007	0-1	-	-
**34**	Masoodpoor, N	2013	6089	27	Kerman	2007-2008	0-1	-	-
**35**	Vakilian, K	2013	20751	3	Semnan	2001-2007	0-1	-	-
**36**	Mohsenzadeh, A	2014	43195	177	Lorestan	2006-2011	0-1	113	64
**37**	Mashhadi Abdolahi, H	2014	22500	46	East Azerbaijan	2004 - 2012	0-1	-	-
**38**	Nikyar, B	2014	18162	138	Golestan	2007- 2009	0-1	76	63
**39**	Khoshhal-Rahdar, F	2014	4235	30	Khuzestan	2013-2014	0-1	-	-
**40**	Hosseini, S	2014	1800	0	Sistan and Balochistan	2012-2012	0-1	-	-
**41**	Amini Nasab, Z	2014	22076	34	South Khorasan	2007- 2012	0-1	-	-
**42**	Bagheri, M.M	2014	2757	74	Tehran	2011 - 2013	0-1	31	43
**43**	Jalali, S.Z	2015	1824	10	Gilan	2011- 2012	0-1	-	-
**44**	Taheri, M	2015	21867	96	Yazd	2012 -2013	0-1	54	42
**45**	Sayehmiri, K	2016	460	3	Ilam	2011-2011	0-1	-	-
**46**	Stone, D. H.	2017	261024	914	East Azerbaijan	2000-2014	0-1	-	-
**47**	Rostamizadeh, L	2017	2738	9	East Azerbaijan	2002-2003	0-1	-	-

ID	First author	Year of publication	Total sample size	Event of CHD	Province	Study period	Age	Male	Female
**48**	Movahedian, A.H	2017	7154	78	Isfahan	2014-2014	0-1	41	37
**49**	Mirfazeli, A	2018	144920	1209	Golestan	2008 - 2013	0-1	-	-
**50**	Safaei Nezhad, A	2018	41265	16	Zanjan	2015-2016	0-1	-	-
**51**	Davari, H.A	2019	156620	56	Isfahan	2014- 2015	0-1	-	-
**52**	Saberi, M	2020	5455	20	Isfahan	2016-2017	0-1	-	-
**53**	Molapour, H	2021	291569	1084	East Azerbaijan	2000 -2019	0-1	-	-
**54**	Tarighat, F	2021	297734	1105	East Azerbaijan	2000-2020	0-1	-	-
**55**	Vafaei, H	2021	1469	24	Fars	2012 -2013	0-1	-	-
**56**	Mohammadzadeh, I	2013	1684	11	Mazandaran	2007-2009	0-1	-	-
**57**	Amiri Simkouii, F	2021	15000	24	yazd	2015-2017	0-1	-	-
**58**	Radvar, M	2022	19941	341	West Azerbaijan	2014-2020	0-1	208	133
**59**	Heidarzadeh, M	2022	347839	144	iran	2019-2021	0-1	-	-
	Heidarzadeh, M	2022	395728	116	iran	2019-2021	0-1	-	-
**60**	Rafati, Sh	2023	160717	309	Yazd	2017-2022	0-1	-	-
**61**	Arandavar, A	2023	5700	3	kermanshah	2018-2022	0-1	-	-
**62**	Asemi-Rad, A	2023	59085	187	Sistan and Baluchestan	2009-2019	0-1	-	-

Table 2

Summary of Specific CHD Subtypes Investigated in the Review

ID*	VSD	ASD	PDA	PS	TOF	AVSD	AS	CoA	HLHS	MR	MVP	PA	TGA	PFO	TR	PR
**1**	-	-	-	-	-	-	-	-	-	-	-	-	-	-	-	-
**2**	-	-	-	-	-	-	-	-	-	-	-	-	-	-	-	-
**3**	-	-	-	-	-	-	-	-	-	-	-	-	-	-	-	-
**4**	-	-	-	-	-	-	-	-	-	-	-	-	-	-	-	-
**5**	22	13	3	-	9	-	1		1	-	-	-	5	-	-	-
**6**	-	-	-	-	-	-	-	-	-	-	-	-	-	-	-	-
**7**	-	-	-	-	-	-	-	-	-	-	-	-	-	-	-	-
**8**	-	-	-	-	-	-	-	-	-	-	-	-	-	-	-	-
**9**	-	-	-	-	-	-	-	-	-	-	-	-	-	-	-	-
**10**	-	-	-	-	-	-	-	-	-	-	-	-	-	-	-	-
**11**	-	-	-	-	-	-	-	-	-	-	-	-	-	-	-	-
**12**	-	-	-	-	-	-	-	-	-	-	-	-	-	-	-	-
**13**	-	-	-	-	-	-	-	-	-	-	-	-	-	-	-	-
**14**	-	-	-	-	-	-	-	-	-	-	-	-	-	-	-	-
**15**	-	-	-	-	-	-	-	-	-	-	-	-	-	-	-	-
**16**	5	-	-	1	2	-	-	-	-	-	-	-	-	-	-	-
**17**	-	-	-	-	-	-	-	-	-	-	-	-	-	-	-	-
**18**	-	-	-	-	-	-	-	-	-	-	-	-	-	-	-	-
**19**	79	166	186	25	165	9	0	39	0	0	0	29	0	0	0	-
**20**	-	-	-	-	-	-	-	-	-	-	-	-	-	-	-	-
**21**	-	-	-	-	-	-	-	-	-	-	-	-	-	-	-	-
**22**	-	-	-	-	-	-	-	-	-	-	-	-	-	-	-	-
**23**	-	-	-	-	2	-	-	-	-	-	-	-	-	-	-	-
**24**	-	-	-	-	-	-	-	-	-	-	-	-	-	-	-	-
**25**	-	-	-	-	-	-	-	-	-	-	-	-	-	-	-	-
**26**	16	4	10	5	0	0	0		0	0	0	0	2	0	0	0
**27**	23	55	68	-	-	-	-	-	-	-	-	-	-	-	-	
**28**	10	31	15	4	2	0	0		0	2	1	0	0	1	2	0
**29**	-	-	-	-	-	-	-	-	-	-	-	-	-	-	-	-
**30**	-	-	-	-	-	-	-	-	-	-	-	-	-	-	-	-
**31**	-	-	-	-	-	-	-	-	-	-	-	-	-	-	-	-
**32**	-	-	-	-	-	-	-	-	-	-	-	-	-	-	-	-
**33**	8	9	5	2	1	0	0		0	0	1	0	0	1	2	1
**34**	-	-	-	-	-	-	-	-	-	-	-	-	-	-	-	-
**35**	-	-	-	-	-	-	-	-	-	-	-	-	-	-	-	-

**36**	78	37	15	8	2	0	6	-	0	0	0	0	7	0	0	
**37**	-	-	-	-	-	-	-	-	-	-	-	-	-	-	-	-
**38**	15	43	15	4	2	0	0		0	2	1	0	1	1	4	-
**39**	-	-	-	-	-	-	-	-	-	-	-	-	-	-	-	-
**40**	-	-	-	-	-	-	-	-	-	-	-	-	-	-	-	-
**41**	-	-	-	-	-	-	-	-	-	-	-	-	-	-	-	-
**42**	16	0	0	0	3	1	2	5	0	0	0	2	3	0	10	-
**43**	-	-	-	-	-	-	-	-	-	-	-	-	-	-	-	-
**44**	16	9	0	4	3	0	0	4	0	0	0	0	0	0	0	
**45**	-	-	-	-	-	-	-	-	-	-	-	-	-	-	-	-
**46**	-	-	-	-	-	-	-	-	-	-	-	-	-	-	-	-
**47**	-	-	-	-	-	-	-	-	-	-	-	-	-	-	-	-
**48**	-	-	11	0	17	0	0		0	0	0	0	6	0	0	-
**49**	-	137	-	-	-	-	-	-	-	-	-	-	-	-	-	-
**50**	-	-	-	-	-	-	-	-	-	-	-	-	-	-	-	-
**51**	-	-	-	-	-	-	-	-	-	-	-	-	-	-	-	-
**52**	-	-	-	-	-	-	-	-	-	-	-	-	-	-	-	-
**53**	-	-	-	-	-	-	-	-	-	-	-	-	-	-	-	-
**54**	-	-	-	-	-	-	-	-	-	-	-	-	-	-	-	-
**55**	-	-	-	-	-	-	-	-	-	-	-	-	-	-	-	-
**56**	-	-	-	-	-	-	-	-	-	-	-	-	-	-	-	-
**57**	8	3	6	0	1	0	0	0	0	0	0	0	0	0	0	-
**58**	102	65	39	2	16	2	2	31	10	0	0	15	26	0	0	-
**59**	-	-	-	-	-	-	-	-	-	-	-	-	-	-	-	-
	-	-	-	-	-	-	-	-	-	-	-	-	-	-	-	-
**60**	-	-	-	-	-	-	-	-	-	-	-	-	-	-	-	-
**61**	-	-	-	-	-	-	-	-	-	-	-	-	-	-	-	-
**62**	19	16	22	0	10	4	5	0	0	0	0	6	0	0	0	-

*The ID in this table corresponds to the order of studies in Table 1.

Table 3

Title of studies included in the review

ID*	Title
**1**	Congenital abnormalities in newborns of consanguineous and nonconsanguineous parents
**2**	Congenital malformations and genetic diseases in Iranian infants
**3**	Statistical Investigation of The Gross Congenital Anomalies in Alive Newborns in Taleghani Hospital
**4**	Prevalence of congenital malformations in neomate born in imam khomeini and shariati hospitals
**5**	Prevalence of congenital heart disorders in neonates hospitalized in Shahid Beheshti Hospital during the years 1996-2000
**6**	A Survey Of Causes And Prevalence Of Congenital Anomalies In Live Bornneonates In Gonabad 22 Bahman Hospital(1373-1380).
**7**	Easily identifiable congental anomalies: Prevalence and risk factors
**8**	Incidence of observable congenital abnormalities among births mirza kochak-khan, imam khomeini and shariatti hospitals (november 2000 to septamber 2001)
**9**	The Study of Incidence of Congenital Anomalies and Relationship Between Anomalies and Personal and Family-Social Factors
**10**	Survey of congenital major malformation in 10,000 newborns
**11**	The prevalence of minor congenital anomalies and normal variations in neonates in bushehr port
**12**	Congenital malformations at a referral hospital in Gorgan, Islamic Republic of Iran
**13**	Prevalence of congenital anomalies in 1000 live births in Javaheri Hospital, Tehran, 2004
**14**	Prevalence of congenital malformations observed in neonates in Shariati Hospital (1381-1383)
**15**	Congenital anomalies in Iran: a cross-sectional study on 1574 cases in the North-West of country
**16**	Pattern of congenital malformations in consanguineous versus nonconsanguineous marriages in Kashan, Islamic Republic of Iran
**17**	Occurrence of congenital anomalies and genetic diseases in a population of Ghazvin Province, Iran: a study of 33380 cases
**18**	Congenital malformations among live births at Arvand Hospital, Ahwaz, Iran-A prospective study
**19**	Prevalence of congenital heart disease in Iran: A clinical study
**20**	The prevalence at birth of overt congenital anomalies in Urmia, Northwestern Iran
**21**	Prevalence of apparent major congenital malformations and some associated factors, in terminated pregnancies in Hajar hospital of Shahrekord, 2005-2006, Iran
**22**	The Incidence of Apparent Congenital Anomalies in Neonates in Mobini Maternity Hospital in Sabzevar, Iran in 2005-6
**23**	The incidence of selected congenital malformations during a two-year period in Tehran, Iran
**24**	Prevalence of congenital malformations in Yazd (Iran)
**25**	The prevalence of congenital malformations and its correlation with consanguineous marriages
**26**	Heart murmur in neonates: how often is it caused by congenital heart disease?
**27**	Early diagnosis and screening of congenital cardiac anomalies
**28**	Prevalence and Pattern of Congenital Heart Disease among Neonates in Gorgan, Northern Iran (2007-2008)
**29**	Congenital anomalies and termination of pregnancy in Iran
**30**	The Frequency Of Congenital Anomalies In Newborns In Two Maternity Hospitals In Bandar Abbas: 2007-2008
**31**	Comparison of congenital abnormalities of infants conceived by assisted reproductive techniques versus infants with natural conception in Tehran
**32**	Prevalence of Congenital Abnormalities and Its Related Risk Factors in Ardabil, Iran, 2011
**33**	Incidence and pattern of congenital malformations in Gorgan-north of Iran

ID*	Title
**34**	Prevalence and pattern of congenital malformations in newborn in rafsanjan, iran (2007-08)
**35**	Frequency of congenital structural anomalies in newborns of Shahroud, Iran
**36**	prevalence and types of congenital heart disease in babies born in the city of Khorramabad (2007- 2011)
**37**	Prevalence of congenital anomalies: a community-based study in the Northwest of Iran
**38**	Ethnical variations in the incidence of congenital heart defects in gorgan, northern iran: a single-center study
**39**	The prevalence of congenital malformations in Dezful-2012
**40**	Race of apparent abnormalities in neonates born in Amir-Almomenin hospital of Sistan
**41**	Demographical condition of neonates with congenital abnormalities under Birjand city health centers during 2007-2012
**42**	Prevalence and etiology of heart murmurs in 2-24-months-old infants Kerman, Iran
**43**	The incidence of obvious congenital abnormalities among the neonates born in Rasht hospitals in 2011
**44**	Population attributable danger of hereditary heart breaks. Risk factors among newborns in Yazd, Iran
**45**	Investigating the prevalence of congenital anomalies and its associated factors in Ilam city
**46**	Uses, Limitations, and Validity of a Registry of Congenital Anomalies in Iran: A Critical Review
**47**	Alteration in incidence and pattern of congenital anomalies among newborns during one decade in Azarshahr, Northwest of Iran
**48**	Congenital Heart Disease: Frequency and the need for intervention on the first year of birth
**49**	Birth Defects in Northern Iran (2008-2013)
**50**	The prevalence of birth defects and related factors in Zanjan city (Northwest, Iran) during 2015-2016
**51**	Major congenital anomalies and associated risk factors in isfahan province, iran, 2014-2015
**52**	Evaluation of Incidence and Main Risk Factors of Major Congenital Anomalies in Hospitals Affiliated with Isfahan University of Medical Sciences during 1395
**53**	Prevalence and time trend of congenital heart defects: a registry-based study in Iran
**54**	Prevalence of Congenital Anomalies in the Northwest of Iran
**55**	Prevalence of single umbilical artery, clinical outcomes and its risk factors: A cross-sectional study
**56**	Prevalence of external congenital malformations in neonates born in Mehregan Hospital, North of Iran
**57**	Comparative study of pulse oximetry, physical examination and echocardiography results in the diagnosis of congenital heart defects in neonates in the first 24 hours of life
**58**	Epidemiology of clinical findings and outcome in neonates with congenital heart disease
**59**	The incidence of congenital anomalies in newborns before and during the Covid-19 pandemic
**60**	Examining the frequency and types of congenital anomalies of newborns born in hospitals in Yazd province
**61**	Investigating the number of congenital anomalies in babies born at Imam Khomeini Hospital in Islamabad Gharb city
**62**	Prevalence of congenital anomalies and related factors in live births in Zahedan, Southeast of Iran: A cross-sectional study

*The ID in this table corresponds to the order of studies in Table 1.
