# Supplementary material for: Spatial Distribution and Birth Prevalence of Congenital Heart Disease in Iran: A Systematic Review and Hierarchical Bayesian Meta-analysis
Source: Int J Health Policy Manag. 2024 May 7;13:7931. doi: 10.34172/ijhpm.2024.7931 (PMC11270618; doi:10.34172/ijhpm.2024.7931)
Supplement: Supplementary file 5 — contains Figures S1-S4 and Table S4. [file ijhpm-13-7931-s005.pdf]

Article title: Spatial Distribution and Birth Prevalence of Congenital Heart Disease in Iran: A Systematic Review and Hierarchical Bayesian Meta-analysis

Journal name: International Journal of Health Policy and Management (IJHPM)

Authors' information: Roghaye Farhadi Hassankiadeh[1], Annette Dobson[2], Somayeh Rahimi[3], Abdollah Jalilian[4], Volker J Schmid[5], Behzad Mahaki[1]*

[1]Department of Biostatistics, School of Health, Kermanshah University of Medical Sciences, Kermanshah, Iran.

[2]School of Public Health, University of Queensland, Brisbane, QLD, Australia.

[3]Department of Clinical Biochemistry, Kermanshah University of Medical Sciences, Kermanshah, Iran.

[4]Department of Statistics, Razi University, Kermanshah, Iran.

[5]Department of Statistics, Ludwig-Maximilians-University, Munich, Germany.

***Correspondence to:** Behzad Mahaki; Email: behzad.mahaki@gmail.com

Citation: Farhadi Hassankiadeh R, Dobson A, Rahimi S, Jalilian A, Schmid VJ, Mahaki B. Spatial distribution and birth prevalence of congenital heart disease in Iran: a systematic review and hierarchical Bayesian meta-analysis. Int J Health Policy Manag. 2024;13:7931. doi:10.34172/ijhpm.2024.7931

Supplementary file 5

Figure S1. Moran's diagram for the standardized prevalence of total CHD per 1000

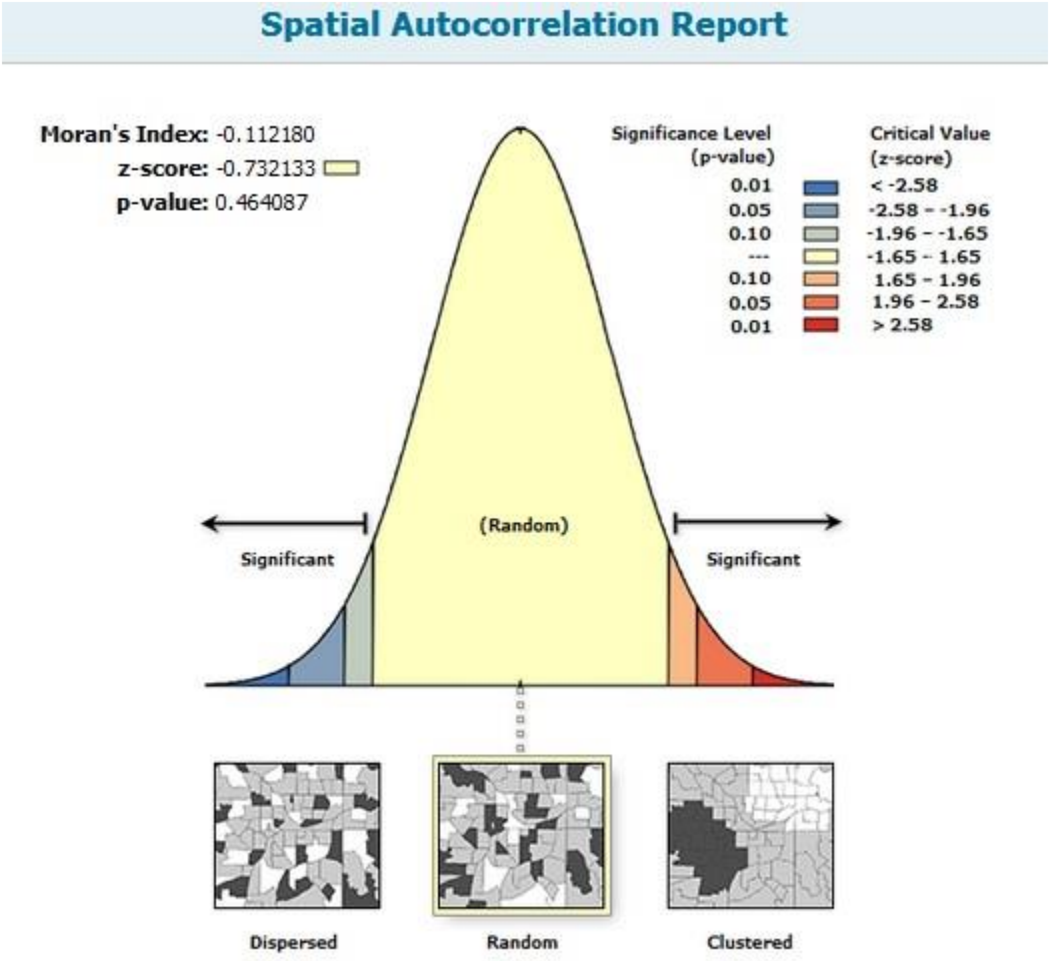

Given the z-score of -0.732132899747, the pattern does not appear to be significantly different than random.

Global Moran's I Summary	
Moran's Index:	-0.112180
Expected Index:	-0.033333
Variance:	0.011598
z-score:	-0.732133
p-value:	0.464087

Figure S1. Forest plot displaying Odds and corresponding 95% credible interval Odds in reported subtypes of CHD studies (17 studies) in Iran

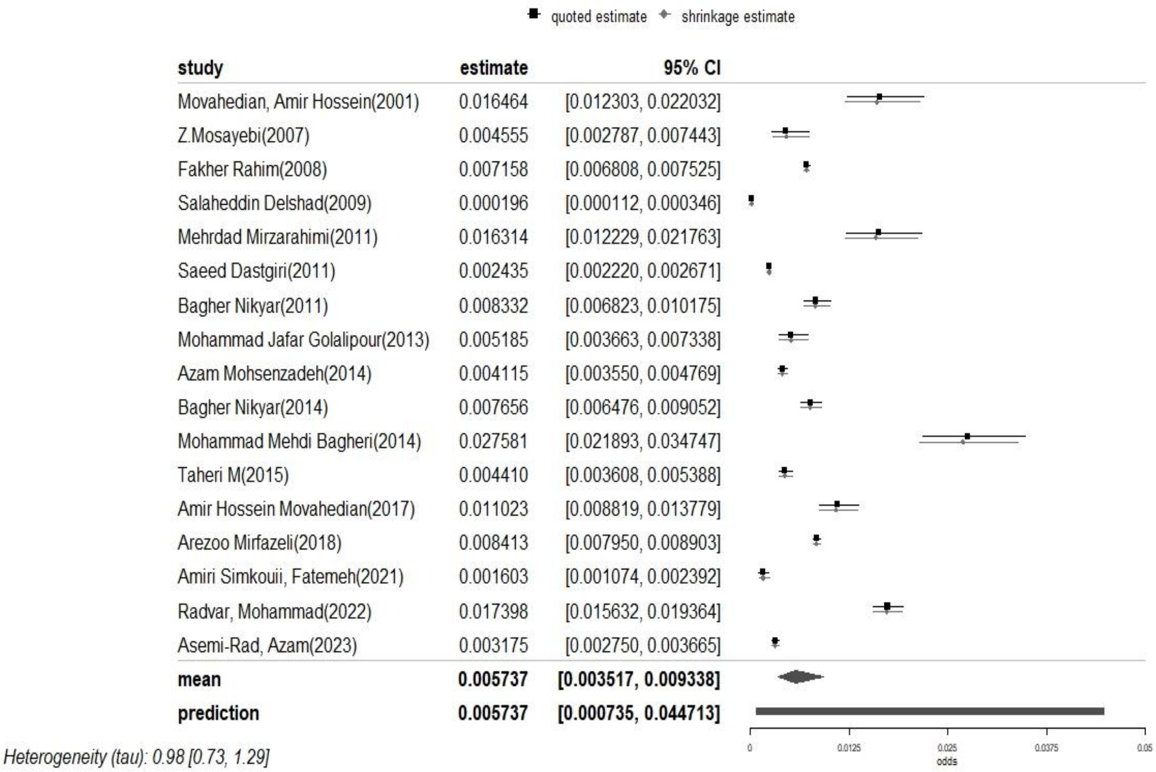

Figure S3. The red lines indicate (approximate) 2-dimensional credible regions, for the joint distribution, Blue lines illustrate the conditional mean effect (Log Odds) as a function of the heterogeneity τ (solid line) with conditional 95% confidence bounds (dashed lines). The green lines show marginal medians and the shortest 95% credible intervals for Log (Odds) and τ. A darker coloring denotes higher posterior density values.

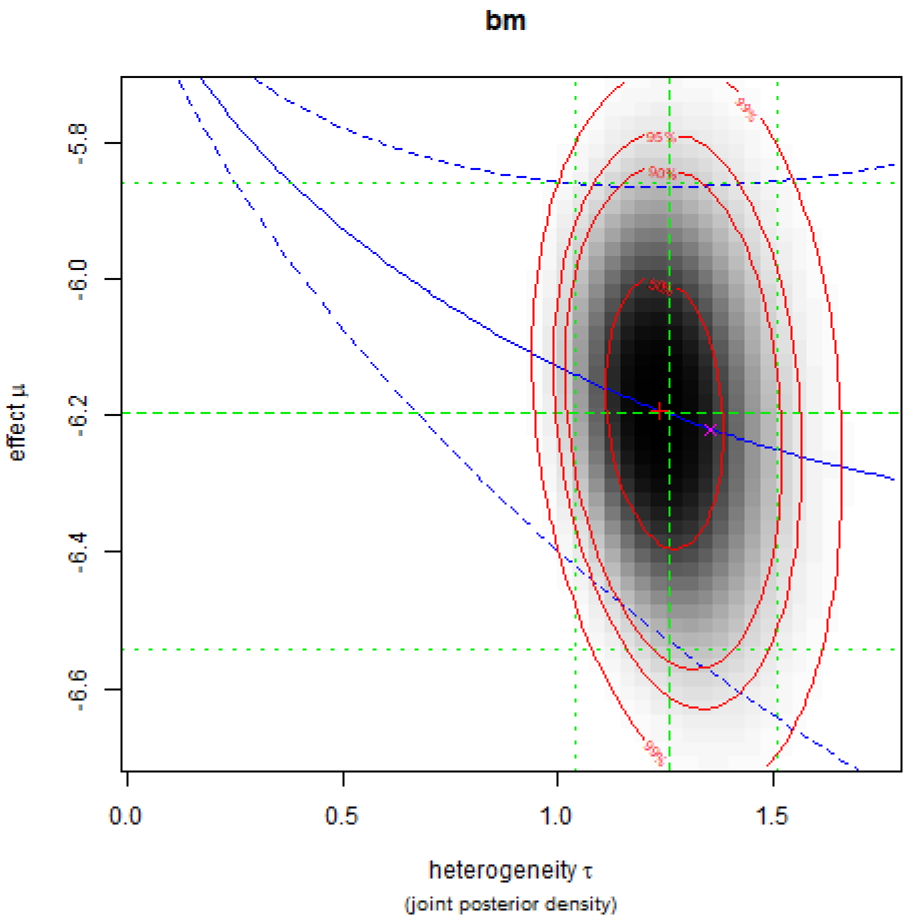

Figure S4. Funnel plot, showing effect estimates (log-odds) vs. their standard errors (σi)

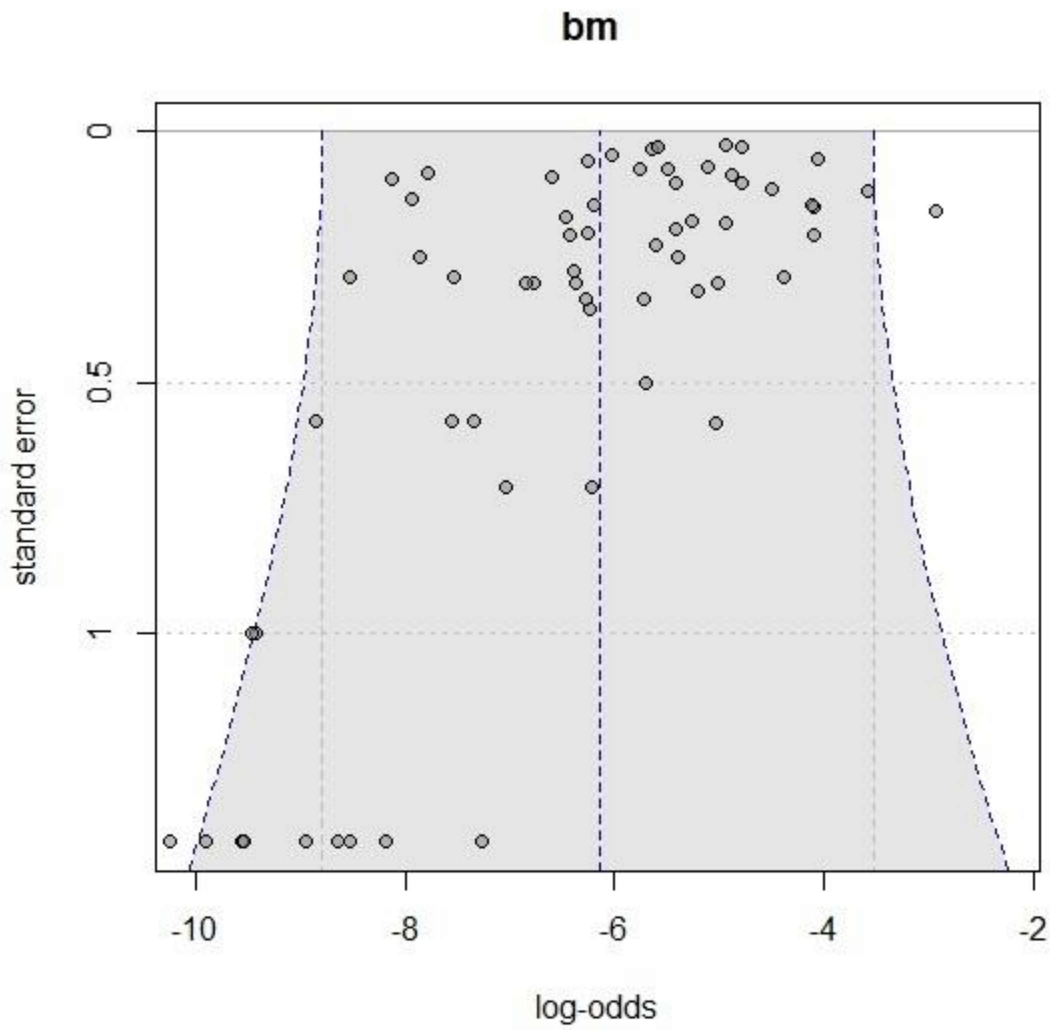

Table S4. Subgroup Analysis for Total and Specific CHD Subtype Birth Prevalence in Iran

variables	levels	Number of Studies	Total	Event	Percentage heterogeneity [I^2]	Pooled log odds [95% Credible interval]	Absolute heterogeneity test τ2 [95% CI]
geographical regions	North	8	205702	1487	0.97	-5.26[-6.07,-4.7]	0.76[0.11,1.4]
	East	6	111295	292	0.83	-6.62[-7.30,-6.07]	0.57[0.23,1.05]
	West	16	1291947	3993	0.99	-6.05[-6.52,-5.60]	0.94[0.64,1.29]
	South	8	251055	1639	0.98	-5.35[-5.79,-4.95]	073[0.44,1.088]
	Center	23	385841	727	0.98	-6.161[-6.71,-5.63]	1.22[0.92,1.57]
length of follow up	<12 month	8	23147	15	0.27	-6.68[-7.60,-5.96]	0.47[0.000,1.078]
	12 to 24 months	25	409211	534	0.97	-6.26[-6.81,-5.72]	1.25[0.94,1.61]
	>24months	23	1729244	7305	0.997	-5.78[-6.41,-5.36]	1.17[0.84,1.55]
Gender	Male	12	242970	720	0.99	-5.84[-6.51,-5.20]	1.10[0.75, 1.5]
	Female	12	242970	580	0.98	-5.99[-6.64,-5.35]	1.07[0.74,1.46]
major subtypes of CHD	VSD	15	671259	417	0.97	-6.84[-7.48,-6.21]	1.16[0.83,1.56]
	ASD	16	816179	588	0.97	-7.09[-7.66,-6.56]	0.95[0.63,1.34]
	PDA	16	678413	395	0.92	-7.3[-7.80,-6.83]	0.77 [0.46, 1.17]
	TOF	15	492763	235	0.91	-7.95 [-8.726,-7.2]	1.127[0.75,1.56]
	PS	12	415838	55	0.64	-8.44[-9.01,-7.88]	0.68[0.32,1.12]
	TGA	13	418678	50	0.81	-8.33[-9.23,-7.50]	1.17[0.70,1.69]
	CoA	13	418678	79	0.80	-8.58[-9.42,-7.82]	0.95[0.55,1.42]
	TR	13	418678	18	0.68	-9.03[-9.99,-8.11]	1.22[0.79,1.70]
	AS	14	477763	16	0.23	-9.21[-9.84,-8.63]	0.46[0.00,1.02]
	PA	14	477763	52	0.65	-8.94[-9.69,-8.25]	0.82[0.43,1.29]
	MR	13	418678	4	0.075	-9.64[-10.36,-8.93]	0.34[0.00, 0.90]
	PH	12	398737	1	0.056	-9.94[-10.76,-9.12]	0.33[0.00,0.91]
	MVP	13	418678	3	0.053	-9.85[-10.58,-9.11]	0.30[0.00,0.85]
	PFO	13	418678	3	0.053	-9.85[-10.58,-9.11]	0.30[0.00,0.85]
	PR	12	398737	2	0.056	-9.94[-10.76,-9.13]	0.33[0.00,0.91]
	HLHS	13	418678	11	0.40	-9.41[-10.32,-8.54]	0.88[0.41,1.41]
	AVSD	14	477763	16	0.075	-9.73[-10.24,-9.20]	0.25[0.00,0.71]
